# How innovations in methodology offer new prospects for volume electron microscopy

**DOI:** 10.1111/jmi.13134

**Published:** 2022-07-27

**Authors:** Arent J. Kievits, Ryan Lane, Elizabeth C. Carroll, Jacob P. Hoogenboom

**Affiliations:** ^1^ Department of Imaging Physics Delft University of Technology Delft The Netherlands

**Keywords:** data management, image analysis, image processing, MB‐SEM, methodology development, volume EM

## Abstract

Detailed knowledge of biological structure has been key in understanding biology at several levels of organisation, from organs to cells and proteins. Volume electron microscopy (volume EM) provides high resolution 3D structural information about tissues on the nanometre scale. However, the throughput rate of conventional electron microscopes has limited the volume size and number of samples that can be imaged. Recent improvements in methodology are currently driving a revolution in volume EM, making possible the structural imaging of whole organs and small organisms. In turn, these recent developments in image acquisition have created or stressed bottlenecks in other parts of the pipeline, like sample preparation, image analysis and data management. While the progress in image analysis is stunning due to the advent of automatic segmentation and server‐based annotation tools, several challenges remain. Here we discuss recent trends in volume EM, emerging methods for increasing throughput and implications for sample preparation, image analysis and data management.

## Lay description

A key concept in biology is that the structure of tissues, cells and their components (cell organelles) often relates to their function. With electron microscopy (EM), it is possible to reveal this structure with nanometre resolution and therefore infer about its function. Electron microscopy of tissues knows a long history of method development, starting in the 1940s. Method development has largely determined the possibilities and scope of electron microscopy. In the 2000s, innovative techniques were developed that allowed routine imaging of tissues in 3D with a higher degree of automation. Nevertheless, conventional electron microscopy techniques remain unsuited for imaging of tissue with nanometre resolution on a millimetre scale because of their low inherent throughput.

Here we analyse trends in volume electron microscopy (EM of tissues in 3D) by reviewing the application, acquisition parameters and data information from over 100 publications in the field. We see an expansion of interest from the conventional applications in neuroscience to other fields, such as cell biology. Additionally, the size of data sets is growing rapidly. From here, we review in detail how certain developments in methodology from the past 10 years have tried to overcome the low acquisition throughput of electron microscopes, by making these techniques more robust during long acquisitions, but also much faster by parallelisation. We find that these new developments have big implications for sample preparation, processing and analysis of the images and data management. We therefore also describe the new developments in these separate domains. We illustrate how novel sample preparation protocols have been developed specifically for larger volumes, how the introduction of machine learning has accelerated automated segmentation of volume EM data and that there is an ongoing transition from local to remote data storage and management. We also touch upon the tools that researchers use to analyse and annotate EM data.

We conclude that the potential of volume EM remains high and the new developments open up possibilities for novel biological studies. We promote the sharing of resources and tools between researchers and institutions to maximise the potential from the new developments in volume electron microscopy.

## 1 INTRODUCTION

Method development is a key factor in accelerating biological discovery. Advances in imaging techniques have fulfilled the desire of biologists to unravel the structure and function of biological systems across a wide spectrum of spatial scales. Electron microscopy (EM) is especially suited for this goal. With its high resolving power, the structure of tissues can be revealed down to the nanoscale. This makes it a useful tool for determining the wiring patterns of neurons,^1^ but also the detailed investigation of cell organelles,^2^ such as microtubules,^3^ mitochondria,^4^ ER^5^ and extracellular vesicles.^6^

The imaging of tissues with EM has a long history of development. The protocols and techniques used to prepare the specimen, initially intended for transmission electron microscopy (TEM), were developed in the early 1940s.^7^ Serial section transmission electron microscopy (ssTEM) was introduced in the 1950s to provide a three‐dimensional context of the tissue.^8^, ^9^ More than half a century later, resin‐embedded tissue samples are still cut into thin sections (albeit much thinner than before) and subsequently imaged with TEM.^10^, ^11^ Until the introduction of computer‐assisted methods in the 1970s, 3D reconstructions of tissue had to be done entirely by hand. For this reason, and because of the extensive manual labour involved in cutting and handling sections, ssTEM applications remained quite limited.^10^

Innovations in the 2000s led to more automated and routine EM techniques for 3D reconstructions of tissue (Table 1), thereby establishing a new research field: volume electron microscopy (volume EM).^12^ As an alternative to ssTEM, serial section electron tomography (ET) was introduced,^13^, ^14^, ^15^ in which a tomographic reconstruction of each serial section is made. Scanning electron microscopes (SEM) allowed for the cutting device to be integrated into the microscope, leading to serial blockface scanning electron microscopy (SBF‐SEM)^16^ and focused‐ion beam scanning electron microscopy (FIB‐SEM).^17^, ^18^ While both offer better axial resolution than ssTEM, they lack the high lateral resolution of TEM and destroy the sample during acquisition. Combining serial sectioning with SEM led to the development of serial section SEM, also known as array tomography (AT).^19^, ^20^ Additionally, Automated Tape‐collecting Lathe UltraMicrotome (ATLUM, later combined into ATUM‐SEM) allows consistent collection and handling of thousands of serial sections.^21^, ^22^

**TABLE 1:** Volume EM methodology

Methodology	Description	Typical *x*, *y* resolution (nm)	Typical *z* resolution (nm)	Typical volume (μm^3^)	Reference
Serial section transmission electron microscopy (ssTEM)	Serial sections are cut with a diamond knife and collected in a water bath, (manually) transferred to a support grid and imaged with transmission electron microscopy. Relatively inexpensive but requires high skill and labour.	4	50	10^3^–10^4^	^10^, ^11^
Electron tomography (ET)	Tomographic reconstruction of 200–1000 nm thin sections by recording at multiple tilt angles. Limited to small volumes.	2–10	2–10	10^2^–10^3^	^13^, ^14^, ^15^
Serial block‐face scanning electron microscopy (SBF‐SEM)	Automated method with microtome inside vacuum chamber. Iteratively a thin layer of material (down to 20 nm) is removed from tissue the block, after which the surface of the block is imaged and the scattered electrons are recorded.	10	30	10^5^–10^7^	^16^
Focused ion beam scanning electron microscopy (FIB‐SEM)	A very thin (2–5 nm) layer of material is iteratively removed by a focused gallium ion beam positioned 45° with respect to the sample, after which the top of the block is imaged. Offers high isotropic resolution.	5	5	10^2^–10^5^	^17^, ^18^
Automated tape‐collecting ultramicrotome SEM (ATUM‐SEM)	Serial sections are automatically cut by a microtome and collected from a water bath on Kapton tape by a computer‐controlled reel‐to‐reel conveyer belt mechanism. Sections are consecutively imaged with SEM.	Flexible	60	10^7^–10^10^	^21^, ^22^
Array tomography (AT)	Ribbons of serial sections are collected on solid surface (silicon wafer, glass) and imaged consecutively with SEM.	4	30, 60	10^4^–10^6^	^19^, ^20^

*Note*: The development and application of these methods is reviewed in Refs. (12), (38), (175) and (176).

Volume EM techniques have been successfully applied in various fields, such as connectomics research (i.e. mapping the connections between neurons),^1^ virology^23^, ^24^ and cell biology.^25^ However, when considering the imaging of ‘large’ volumes (>10^6^ μm^3^), the aforementioned EM approaches quickly run into their limits as the throughput of modern electron microscopes remains low. The imaging and reconstruction of larger volumes can take up to several months or years in some cases.^26^, ^27^, ^28^, ^29^ Additional challenges include the increased risk of acquisition errors and loss of material during long acquisitions, generation of very large data sets (hundreds of terabytes) and enormous manual annotation efforts.^30^, ^31^ As a consequence, every volume EM study is a tradeoff between resolution, acquisition speed, long‐term system stability and the effort needed in annotation.

Despite these challenges, new light is shining on the volume EM field. Powerful electron microscopes with unrivalled acquisition speeds have recently made their entrance.^32^, ^33^ At the same time, the throughput of existing methods has increased significantly by advancements in software and hardware.^27^, ^29^, ^34^, ^35^, ^36^, ^37^ Years of imaging with conventional systems could now in principle be reduced to a few weeks. In this review, we analyse trends in volume EM and focus on the specific improvements in methodology that relieve the bottleneck in throughput of electron microscopes. We then discuss the implications of these developments for sample preparation, image analysis and data management respectively.

## 2 TRENDS IN VOLUME ELECTRON MICROSCOPY

**FIGURE 1:**
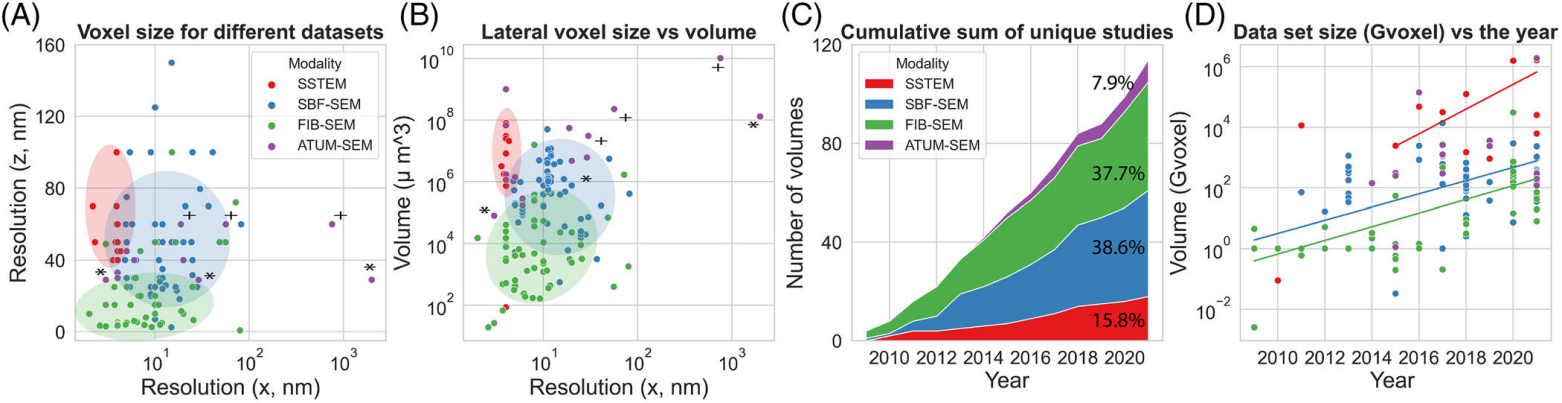
Overview of VEM studies from 2009–2021 reporting voxel resolution, method, volume and data set size. (A) Voxel size (*x* vs. *z*) and (B) volume size for all data sets. Ellipses indicate application regimes. (C) Cumulative sum of studies per method. (d) Increase in data set size per year. All data points represent a single data set, except those marked by * and +, which are targeted re‐acquisitions from Kasthuri et al.^72^ and Hildebrand et al.^26^ respectively

To distil general trends in volume EM, we summarised relevant statistics from a pool of over 200 EM volumes from 115 unique studies (Figure 1) conducted between 2009–2021, including those covered in earlier reviews.^12^, ^38^, ^39^ It is inevitable that certain statistics are missing from a number of studies as certain data set parameters such as volume size, voxel resolution and data set size are not consistently reported and have yet to be standardised (connectomics studies being an exception^39^).

We searched and grouped studies based on the used techniques: serial section transmission electron microscopy (ssTEM), serial blockface scanning electron microscopy (SBF‐SEM), focused ion beam scanning electron microscopy (FIB‐SEM) or automated tape‐collecting ultramicrotome SEM (ATUM‐SEM). The latter can be considered as a subset of array tomography, but whereas array tomography is also frequently associated with light microscopy, ATUM‐SEM is a more dedicated volume EM technique. Certain application regimes can be distinguished (Figure 1A and B). FIB‐SEM is clearly in the high resolution but low volume regime, whereas ssTEM studies typically target large volumes with high lateral resolution – suitable for use in connectomics. ATUM‐SEM and ssTEM show great flexibility in the volume size and resolution, because they allow re‐imaging of parts of the sample with different settings. SBF‐SEM is a ‘mid‐range’ method, covering a volume range from roughly 10^4^ to 10^7^ μm^3^. Additionally, the number of volume EM studies is increasing at a steady rate. The majority of studies has been conducted using SBF‐SEM or FIB‐SEM (Figure 1C). Although the number of studies per year varies quite a bit, there is a clear trend towards bigger data sets (Figure 1D).

The push towards larger volumes can be explained by connectomics research. Scientists have fully reconstructed the nervous systems and determined the connectomes of small organisms, and partially in bigger organisms (Table 2). From the smallest (*C. elegans* larval brain^40^) to the largest volume (mouse visual cortex^41^) at full resolution, the size difference is more than five orders of magnitude. While connectomics research can be considered a driver for innovation in the field, the application of volume EM is linked to several other research fields.^24^, ^42^, ^43^, ^44^, ^45^, ^46^ We found over 110 distinct applications in 23 different organisms, including animals (and their larval stages), plants, bacteria and cell lines (Supplementary Table S1). Some studies feature reconstructions of single‐cell organisms and small organisms such as the budding yeast (*S. cerevisiae*),^47^ parasite *Trypanosoma brucei* ^48^ or the ringed worm (*P. dumerilii*) at 6 days post‐fertilisation.^49^ Some of the larger samples are intersegmental vessels and dorsal‐lateral lanastomotic vessels in zebrafish embyos^50^ (FIB‐SEM), root tips of the barrelclover (*M. truncatula*),^38^ (SBF‐SEM), human and mouse fibrous connective tissue^51^ (SBF‐SEM) and mouse liver tissue^52^ (FIB‐SEM).

**TABLE 2:** Relevant imaged volumes in connectomics research

Organism/species	Stage	Tissue	Method	Connectome determined	Volume size (μm3)	Data set size (TB)	Reference(s)
*C. elegans (Roundworm)*	Larva L3	Hermaphrodite full brain	SSTEM	Fully	15,707	–	^40^
	Larva L2	Hermaphrodite full brain	ATUM‐SEM	Fully	15,351	–	^40^
	Larva L1	Hermaphrodite full brain	ATUM‐SEM	Fully	5148	–	^40^
	Larva L1	Hermaphrodite full brain	SSTEM	Fully	4251	–	^40^
	Larva L1	Hermaphrodite full brain	ATUM‐SEM	Fully	2989	–	^40^
	Larva L1	Hermaphrodite full brain	ATUM‐SEM	Fully	4536	–	^40^
	Adult	Hermaphrodite CNS	SSTEM	Fully	–	–	^177^
	Adult	Hermaphrodite full brain	SSTEM	Fully	52,382	–	^40^
	Adult	Hermaphrodite full brain	ATUM‐SEM	Fully	73,850	–	^40^
	Adult	Male CNS	SSTEM	Fully	724,776	–	^178^
*C. intestinalis (Sea squirt)*	Larva	Central nervous system	SSTEM	Fully	*–*	*–*	^179^
*D. melanogaster (Fruit fly)*	Larva	Central nervous system	SSTEM	Partly	1,755,837	–	^180^
	Adult	Central brain	FIB‐SEM	Fully	1,562,5000	26	^28^
	Adult	Full brain	SSTEM	Partly	79,150,500	106	^27^, ^132^
	Adult	Mushroom body	FIB‐SEM	Fully	240,000	3.8	^181^
	Adult	Olfactory system	SSTEM	Partly	250,000,000	50	^75^
	Adult	Ventral nerve cord	SSTEM	Partly	21,000,000	172.6	^37^
*D. rerio (Zebrafish)*	Larva	Entire brain	ATUM‐SEM	Partly	10,200,000,000	4.4	^26^
	Larva	Hindbrain	ATUM‐SEM	Partly	1,404,480	–	^182^
	Adult	Spinal segment	SBF‐SEM	Fully	11,716,726	–	^183^
*H. Sapiens (Human)*	Adult	Cerebral cortex	ATUM‐SEM	Partly	1,000,000,000	1400	^41^
*M. musculus (Mouse)*	Adult	Cochlea (inner hair cells)	SBF‐SEM	Partly	6,841,300	0.194	^184^
	Adult	Cochlea (inner hair cells)	SBF‐SEM	Partly	7,796,872	–	^184^
	Adult	Cortex layer 4	SBF‐SEM	Fully	542,510	–	^31^
	Adult	Neo cortex	SSTEM	–	1,000,000,000	2000	^29^
	Adult	Neo cortex	ATUM‐SEM	Partly	130,000,000	0.3	^72^
	Adult	Somatosensory cortex	ATUM‐SEM	Partly	30,500,000	0.14	^185^
	Adult	Visual cortex	SSTEM	partly	30,375,000	100	^65^
	Adult	Visual cortex	SSTEM	Partly	8,190,000	–	^35^
	Adult	Visual cortex	SSTEM	Partly	1,049,022,720	2000	^45^
	Adult	Visual Thalamus	ATUM‐SEM	Partly	67,200,000	100	^73^

The number of volumes with high isotropic resolution is also increasing. Abnormalities in cell organelle structure and function are implicated in the development of diseases, which can be studied in detail with FIB‐SEM. Volume EM studies with FIB‐SEM have resulted in high resolution 3D reconstructions including (but not limited to) HeLa cells, T cells and macrophages,^2^, ^43^, ^53^ cancer cells,^54^ mouse primary beta cells^3^ and COS‐7 cells.^55^ Moreover, studies have been performed on human cardiac telocytes,^56^ mouse liver tissue^52^ and lung alveolar epithelium.^57^ Studies of abnormal ultrastructure are emerging, including breast carcinoma and pancreatic adenocarcinoma.^46^

In short, volume EM applications have expanded well beyond the scope of connectomics, and the various techniques can be demarcated into distinct application regimes. Data sets are increasing in size and becoming more diverse. We will show later that some trends can be attributed to specific developments in hardware (Section 3), while others may be a result of the general increase in popularity of volume EM and access to better equipment. We will now layout the new developments in methodology which have contributed to some of the trends that are described here.

## 3 IMAGING OF LARGER BIOLOGICAL VOLUMES

**FIGURE 2:**
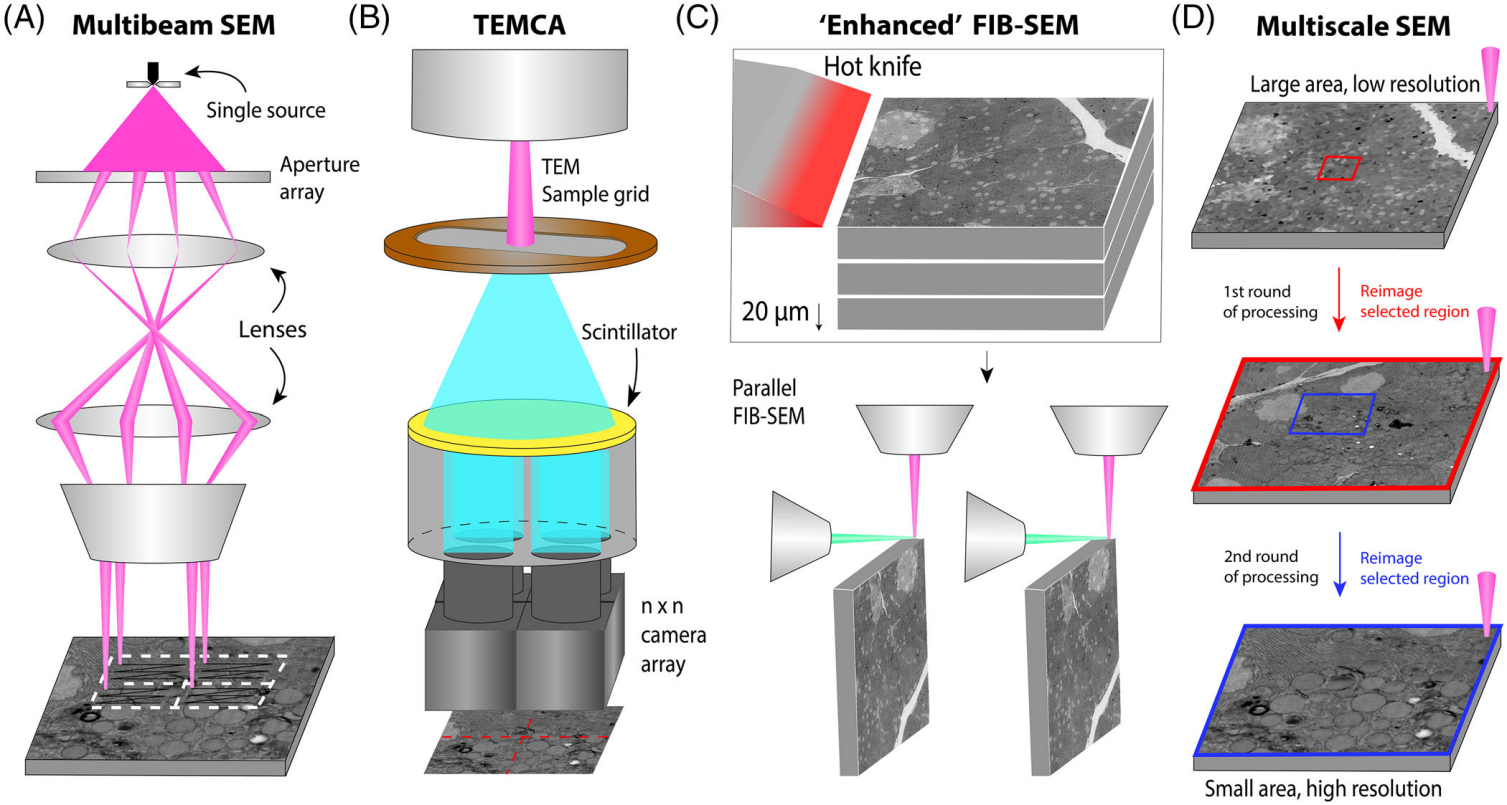
Four different methods for scaling up volume EM studies: (A) Multibeam electron microscopy. Throughput is increased by using multiple beams in parallel. (B) TEMCA^35^ and AutoTEM^29^ principle. Throughput increase by multiple parallel cameras to enlarge the field of view of the microscope. (C) ‘Enhanced FIB‐SEM’: ultrathick sectioning^34^ is applied to a sample that is too thick to be handled by a single FIB‐SEM. Throughput increase is achieved by higher system stability and using multiple FIB‐SEMs in parallel. (D) Single‐beam multiscale EM. ‘Increased’ throughput by scanning a large area at low magnification followed by multiple rounds of targeted acquisition at higher resolution

A major feat in volume EM would be to routinely image volumes larger than 1 mm^3^ at nanometre resolution in a few months. Achieving this is not only a matter of improving speed; instrumentation must be able to robustly image thousands of tissue sections or slices for extended periods with minimal intervention. Therefore, instrumentation development has focused not only on increasing imaging speed, but also robustness and automation. New developments can roughly be divided into four groups: (1) parallelisation by multiple beams, (2) parallelisation by multiple cameras, (3) parallel processing in block‐face imaging and (4) re‐imaging of volumes at different resolution scales (Figure 2).

### 3.1 Multiple scanning beams in parallel

The imaging speed in (volume) EM is limited by the minimum signal‐to‐noise ratio (SNR) needed to make biological features sufficiently visible against a noisy background. The SNR is influenced by the exposure time, beam current, sample contrast and detection efficiency.^32^ In order to achieve faster imaging, it seems straightforward to increase the beam current. However, this leads to lower resolution due to increased coulomb interactions and can go at the cost of sample charging, inducing sample drift and artefacts. A workaround would be to use multiple beams in parallel. This idea has led to the development of multibeam scanning electron microscopy (MB‐SEM).^32^, ^33^ An MB‐SEM scans the sample simultaneously with an array of beamlets produced by a single electron source (Figure 2A), increasing the acquisition speed proportionally to the number of beamlets with theoretically no compromise on resolution compared to single‐beam SEM. Multiple concepts have been developed for multibeam electron microscopy with different source and column configurations, beam array sizes and detector systems.^32^, ^33^, ^58^, ^59^

**FIGURE 3:**
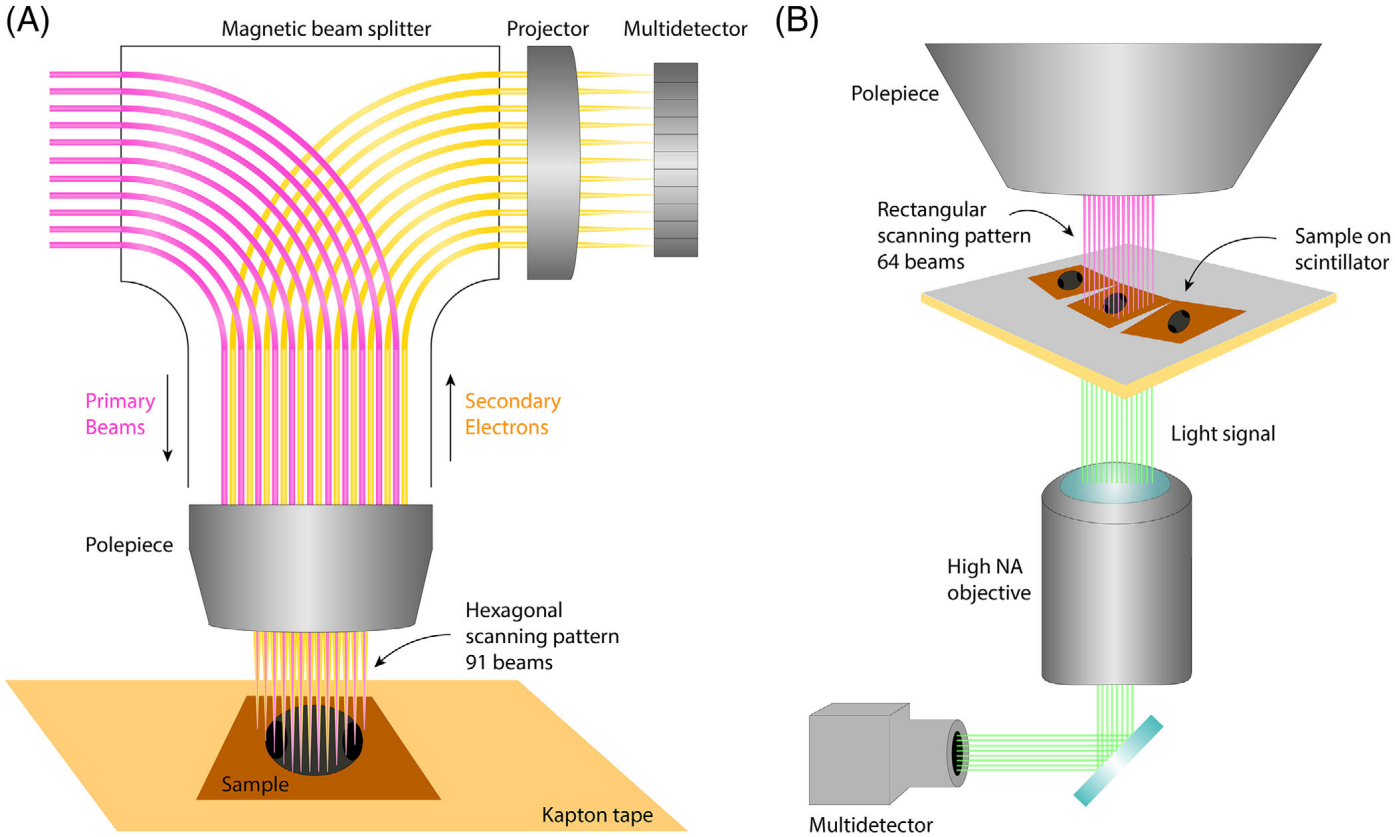
Different multibeam systems. (A) Zeiss MultiSEM,^32^ which makes use of secondary electron detection by a multidetector. The primary beams and detected electrons are separated by a magnetic beam splitter. (B) Delft Multibeam (FAST‐EM).^33^, ^174^ Instead of secondary electron detection, this multibeam system detects transmitted electrons via conversion to a light signal by a scintillator located directly under the sample

The first commercially available MB‐SEM (MultiSEM) was released in 2015,^32^, ^60^ producing 61 or 91 beams in a hexagonal pattern (Figure 3A). The primary beams originate from a single source and go through a single column, where they are separated from secondary electron signals by a magnetic beam splitter. Each secondary electron signal is lead to a dedicated secondary electron detector. The number of beams can be increased without changing the primary design; a 331 beam version has subsequently been developed,^61^ though it is not yet commercially available.

A single‐source 196‐beam MB‐SEM was developed at Delft University of Technology.^58^ This model employs transmission electron detection instead of direct secondary electron detection. The sample is placed on a luminescent material (scintillator) coated with a thin conductive layer which converts the electrons to photons. The light beams are then imaged onto a detector array^33^, ^62^ (Figure 3B). A dedicated 64‐beam MB‐SEM system (FAST‐EM) using this technology has recently been commercialised.

While MB‐SEM is not yet widely applied in volume EM, the first study results are impressive. A large‐scale (2D) study of mouse and marmoset brain tissue was performed.^63^ Another study revealed for the first time the complex structure of the chicken retina.^64^ The latest result is a 1.4 petabyte data set of human cerebral cortex acquired in 326 days,^41^ which was fully segmented using automated methods discussed later (Section 5). These pioneering studies indicate great potential.

### 3.2 Multiple cameras: TEMCA and AutoTEM

Transmission electron microscopy is inherently parallel compared to scanning electron microscopy. However, it is slowed down significantly by sample stage movement, detector readout time and sample grid replacement. TEM camera array (TEMCA, Figure 2B) was developed to improve the throughput of transmission electron microscopes.^35^, ^65^ The field of view of the TEM is increased by using a 2 × 2 array of high‐speed CCD (charge‐coupled device) cameras coupled to lenses, connected to an extended vacuum column. To further improve throughput, Zheng et al.^27^ built two second‐generation TEMCA systems (TEMCA2), equipped with four CMOS cameras, a custom piezo‐driven fast stage and an automated transport and positioning system, which allow unsupervised sample loading and imaging for extended periods. Together, these innovations allow 40× faster imaging than conventional TEM. The TEMCA2 design was used to image a full adult fruit fly brain in 16 months.^27^

The TEMCA2 has inspired the development of a further automated TEM system, autoTEM.^29^ AutoTEM consists of 6 parallel TEMCA‐inspired systems with a summed burst acquisition rate of 3 Gpixel/s and a net rate of 600 Mpixel/s. A new nano‐positioning sample stage offers fast montaging of large areas.^37^, ^66^ The sections are loaded onto a new aluminium‐coated polyimide tape with regularly spaced TEM‐grid‐resembling holes (GridTape^37^), which enables section collection with ATUM. The implementation of a new reel‐to‐reel tape translation system allows loading and selection of 5500 sections per vacuum cycle. With AutoTEM, two 1 mm^3^ volumes of mouse neocortex and primary visual cortex were imaged in about 6 months, resulting in two petabyte data sets.^29^, ^45^ Additionally, a TEMCA system upgraded with GridTape was used to reconstruct the ventral nerve cord of a female fruit fly, resulting in a 172.6 terabyte data set.^37^

### 3.3 Parallel processing in block‐face imaging

While serial block‐face methods are used in the majority of volume EM studies (Figure 1C), increasing their throughput is not trivial. So far, block‐face methods remain incompatible with MB‐SEM. While acquisition in SBF‐SEM is highly automated, the samples are prone to charging and sensitive to beam dose. Solutions to these problems are described later (Section 4). An even bigger challenge is increasing the low throughput of FIB‐SEM, which is a result of slow FIB‐milling speeds and limited robustness of FIB‐SEM systems.

#### 3.3.1 Parallel ‘Enhanced’ FIB‐SEM

To make FIB‐SEM systems more suitable for volume EM, Xu et al.^36^ developed ‘Enhanced FIB‐SEM’ (Figure 2C). Enhanced FIB‐SEM expands the scope of FIB‐SEM from 1000 μm^3^ to 3 × 10^7^ μm^3^ – four orders of magnitude. FIB‐milling limits the sample thickness to about 100 m in the milling direction because it introduces streaks and waves of thickness variation. A solution was found in smooth ‘ultrathick’ partitioning of tissue volumes.^34^ Resin embedded tissue is cut into multiple chunks of 20 μm with a hot ultrasonic vibrating diamond knife to reduce distortions and slips. The chunks can then be imaged separately and stitched together. Additionally, signal detection is improved by a small positive stage bias that filters out secondary electrons, allowing efficient backscatter detection by an in‐column detector. The working distance is reduced by repositioning the FIB column to be 90° from the SEM column. Lastly, a special closed loop control system is used to maintain ion beam stability and allow seamless restarts. Two ‘enhanced’ FIB‐SEMs were employed in a study that reconstructed the connectome of the fruit fly central brain.^28^

#### 3.3.2 Alternative milling approaches

Other milling approaches could potentially offer higher throughput than conventional gallium ion FIB, including gas cluster ion beam (GCIB),^67^ broad ion beam (BIB)^68^ and plasma focused ion beam (PFIB) milling.^69^ In GCIB‐SEM, 500 nm to 1 μm thick sections are collected from the sample. These sections are pre‐irradiated with the SEM to reduce charging, followed by milling at 30° with clusters of low energy argon ions. Volumes with 10 nm isotropic resolution were acquired, but full integration with high‐throughput SEM (i.e. MB‐SEM) has yet to be demonstrated. With BIB, large areas (up to several mm) can be milled while simultaneously offering a sputter rate up to five times higher than in gallium ion FIB.^70^ Milling and imaging of liver and mouse brain tissue has been demonstrated with an integrated BIB‐SEM system, although not with high isotropic resolution as in FIB‐SEM. Xenon ion PFIB offers low damage milling compared to gallium FIB with 20–60× faster rates,^69^ but has not been widely adopted for biological samples. Oxygen has also been proposed as an alternative ion species with greater resin compatibility and similar potential gains in throughput.^71^

### 3.4 Targeted reimaging with multiscale EM

Unlike block‐face methods, serial sectioning methods like ssTEM and ATUM‐SEM allow re‐imaging of tissue sections. This has inspired some researchers to use a multiresolution approach when imaging large volumes with ATUM‐SEM^26^, ^72^ (Figure 2D), to limit acquisition time. After recording the complete volume at low magnification, targeted regions of interest can be re‐imaged at higher magnification to reveal smaller features. In connectomics, this is convenient because most neuronal branches can be traced at lower resolutions while only some parts are needed in high resolution for completion.^73^ Moreover, the different data sets can be registered and combined into a multiresolution data set.

Another multiresolution approach combines ATUM with targeted high isotropic resolution FIB‐SEM, a new method called ‘multiscale ATUM‐FIB microscopy’.^74^ In ATUM‐FIB, serial sectioning of tissue into ‘semithick’ 2–10 μm sections is done first to create a library by attaching them onto glass slides that can be imaged with light microscopy. Then, they are remounted onto silicon wafers for serial section SEM to identify regions of interest to target with high resolution FIB‐SEM.

## 4 SAMPLE PREPARATION FOR LARGE VOLUMES

The success of a volume EM study is ultimately determined by the quality of sample preparation. It is inherently difficult to prepare biological samples for electron microscopy; they should be compatible with staining and residing in vacuum, have sufficient and homogeneous contrast and be resistant to sectioning and beam irradiation. While sample preparation protocols are typically designed for a specific target species or tissue type, they follow roughly the same steps: (1) fixation with aldehydes, (2) staining with heavy metals such as osmium, uranium and lead, (3) tissue dehydration and (4) resin embedding, followed by sectioning. The whole procedure, including sectioning, can take up to a few weeks per sample.^29^, ^45^ With acquisition times of large volumes being significantly reduced by emerging new methods (Section 3), further optimisation of sample preparation protocols with respect to throughput becomes increasingly important. We describe next the implications of the throughput increase in acquisition on sample preparation.

### 4.1 Approaches in fixation and staining

**FIGURE 4:**
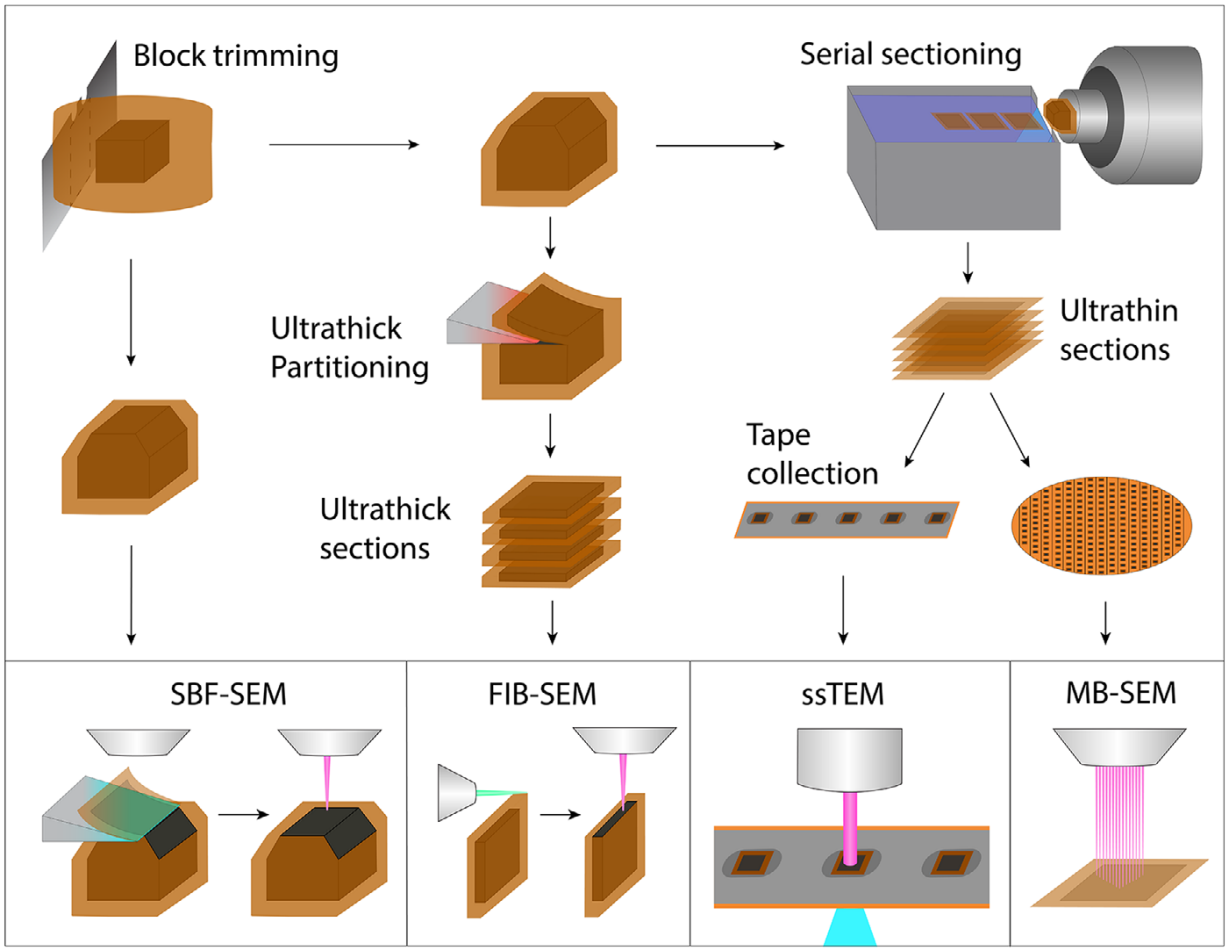
Sample preparation steps for high‐throughput volume EM. Next to improvements in en bloc staining, ultrathick partitioning was introduced in FIB‐SEM and tape collection in ssTEM. Figure not to scale

Sample preparation protocols have been modified to allow for higher throughput acquisition methods (Figure 4) as well as for homogeneity of fixation and staining for larger than before sample volumes. To provide homogeneous preservation of the tissue, it is either dissected before fixation,^27^, ^28^, ^75^ or perfused with a fixative solution before dissection.^26^, ^29^, ^31^, ^65^, ^72^, ^73^, ^76^ To further promote diffusion of the fixative into the sample, the surrounding skin can be removed.^26^ Fixation is typically followed by *en bloc* staining, in which the sample is submerged into one or more solutions of (different) heavy metal compounds to increase electron scattering. In traditional serial section TEM, samples are typically treated twice: first *en bloc*, then by *post staining* the ultrathin sections to enhance the contrast. However, post‐staining is laborious, prone to contamination and incompatible with block‐face techniques.

Hence, volume EM sample preparation protocols have been designed to optimise *en bloc* staining. The traditional osmium‐thiocarbohydrazide‐osmium (OTO) protocol,^77^ in which thiocarbohydrazide acts as a bridging agent for osmium tetraoxide to crosslink and stain cell membranes, typically leads to inhomogeneous staining for larger volumes. By addition of potassium ferri‐ or ferrocyanide, the osmium can be reduced to make it more reactive (reduced OTO or rOTO^78^). While this improves contrast and thereby allows for lower dwell time, it still has a limited penetration depth (∼200 μm) and weakens large tissue samples due to the formation of nitrogen bubbles. The OTO protocol was therefore modified further by separating the osmium and ferrocyanide treatment steps.^79^ This allows the osmium to penetrate deeply into the tissue, after which it is reduced to allow for deeper staining. A variant on this protocol adds formamide during the reducing osmium step and replaces thiocarbohydrazide by pyrogallol, which prevents nitrogen bubble formation.^80^ This protocol was further optimised to reduce the long incubation times,^81^ thus allowing both homogeneous, strong fixation and staining as well as faster sample preparation.

### 4.2 Sectioning of large volumes

Three out of four emerging volume EM techniques discussed earlier rely on serial sectioning, motivating the need for reliable sectioning approaches. Cutting and collecting (thousands of) ultrathin serial sections is a delicate process; many factors affect the consistency and continuity. An inherent issue is that interruptions are needed to resharpen or replace the knife, which impair sectioning quality as the knife needs to be repositioned. A closed‐loop repositioning system as introduced in FIB‐SEM may offer a solution. Another issue is section collection. Multiple tools have been developed that simplify the handling and collection of moderate amounts of sections,^82^, ^83^, ^84^ but for larger amounts automated collection (ATUM) is currently the only viable option. The collection tape of ATUM has a low packing density (∼200 sections per metre) and needs to be carbon‐coated for conductivity. Intrinsically conductive alternatives such as carbon‐nanotube tape,^85^ on the contrary, need plasma treatment for hydrophilisation and manual grounding. In order to increase the packing density of sections, Templier^86^ introduced MagC, in which the tissue block is glued to a magnetic resin, which allows magnetic collection of the sections directly onto wafers.

### 4.3 Charge‐compensation and artefact reduction

Artefacts created during sample preparation and acquisition increase the difficulty of reconstructing volumes with automated image processing methods.^28^, ^31^, ^41^ There are several ways in which these artefacts can be reduced. One way is focal charge compensation, in which surface charges are neutralised by local injection and ionisation of nitrogen gas onto the sample.^87^ Additionally, the conductivity can be increased by coating the sample with a thin metallic film prior to each cycle of imaging.^88^ The embedding material can also be made more conductive, either with a metallic particle filler^89^ or adding carbon powder.^90^, ^91^ New types of resins can also offer higher contrast with low stain concentrations, offering a way to reduce artefacts introduced by staining.^92^ Lastly, the sample can be embedded within another biological sample^26^ to improve stability of the tissue block and prevent shrinkage and deformation.

## 5 IMAGE PROCESSING AND ANALYSIS IN VOLUME EM

**FIGURE 5:**
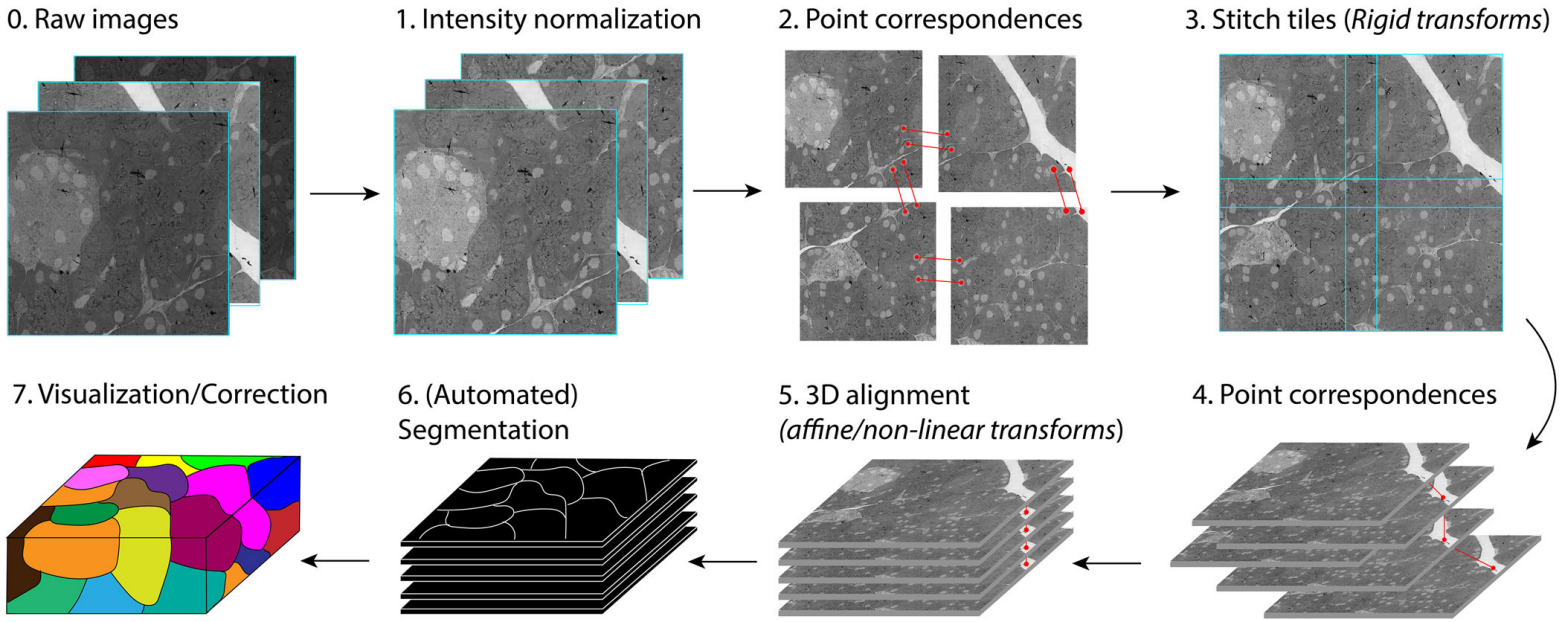
Image processing and analysis workflow. First, the images are normalised. Next, images that belong to one section are stitched with help of point‐pair matches and transformations. Similarly, the composite images of the sections are 3D aligned. When the 3D volume has been solved, the segmentation can be performed (automatically). Segmentation is followed by annotation and visualisation. The data can then be interpreted and analysed

Image processing and analysis of volume EM data sets are nontrivial tasks due to their size and complex nature. Roughly speaking, the steps in image processing are intensity normalisation, 2D stitching and 3D alignment, while image analysis concerns the annotation (labelling individual biological features in the data set) and segmentation (assigning every pixel or voxel to a class) of the reconstructed volume to extract biological information (Figure 5). The computer algorithms that handle these tasks have to overcome difficulties such as variable intensity and contrast, sample drift, missing or low‐quality data, and imaging artefacts introduced by sample preparation, sectioning (shear, distortion), pickup, inhomogeneous staining and beam damage. The throughput increase also poses additional challenges for image analysis. Manual segmentation of volumes, already a time‐consuming process for small data sets, becomes impractical for large data sets. We will illustrate the steps in image processing and analysis while discussing the state of the art approaches and methods.

### 5.1 Stitching of large FOVs and 3D alignment

When a region of interest (ROI) is larger than the field of view (FOV) of the microscope at the desired magnification, multiple FOVs are acquired with a small overlap and digitally stitched together to reconstruct the whole ROI (commonly referred to as a *montage* or *mosaic*). There are multiple algorithms for stitching, which generally work for all EM techniques considered here. The simplest in terms of computational complexity is phase correlation, which computes the translation between two overlapping image tiles based on the normalised cross‐correlation.^93^ However, phase correlation does not take into account affine transformations and only allows for local optimisation. A more robust approach is to find local point‐pair correspondences between images with a feature detection algorithm, such as the scale‐invariant feature transform (SIFT)^94^ or speeded up robust features (SURF).^95^ Both algorithms use scale‐space representations – consisting of increasingly downsampled versions of the images – to find scale‐invariant features. Corresponding point‐pair matches are selected using robust sampling methods (RANSAC)^96^ and from these the affine transformations are determined to generate a globally optimised alignment.

The same algorithms can be used to align individual mosaics in 3D. First, each mosaic is downsampled and roughly aligned to its neighbouring layers. This is then refined by extracting and matching point‐pair correspondences between neighbouring tiles in different layers. In serial block‐face methods (FIB‐SEM and SBF‐SEM), only subtle refinement may be needed as the FOV is inherently highly similar between adjacent slices. Alignment of ssTEM and ATUM‐SEM is more complicated as it requires significant corrections for rotation and non‐linear distortion compared to block‐face data sets.

There are several dedicated software packages for stitching and 3D alignment, including AlignTK,^35^, ^65^ NCR tools,^97^ StackReg^98^ and Big Feature Aligner (BigFeta).^99^ Popular tools that can perform EM image registration are TrakEM2^100^ – implemented in Fiji (ImageJ), popular among bioimage analysts – IMOD^101^ and Microscopy Image Browser (MIB).^102^ Lastly, a novel approach was developed for multiscale EM alignment, known as signal whitening Fourier transform (SWiFT‐IR),^103^ in which modulated Fourier transform amplitudes produce more robust image matching.

### 5.2 Manual annotation and segmentation

After the volume is reconstructed, the next step is to extract biological information from the data. To quantify the morphology of tissues, cells and cell organelles, their 3D structure has to be annotated and segmented. Successful interpretation of biological EM images is time consuming and requires training in anatomy. It was recently estimated that it would take up to 60 years to manually segment each organelle in a single cell by hand.^2^ Nevertheless, volume EM studies still rely on manual or semi‐automatic segmentation and annotation. Usually, only a fraction of the entire volume is annotated by hand (*sparse annotation*) to reduce the workload. These annotations can be used as training data for machine learning algorithms to process the whole volume in an automated fashion.

#### 5.2.1 Voxel painting and neuron tracing

The most straightforward method is inspecting and labelling voxels with the help of (web‐based) software. These applications allow browsing through a volume and facilitate tracing of cells, membranes, cell organelles or other features of interest. Groups of voxels can be assigned a label with brush or bucket tools. Neurons are frequently annotated by a centre‐line tracing (*skeleton*). Software tools often support multiple approaches. To get better accuracy, tracings can be proofread by an additional annotator. In large connectomics studies, typically a team of multiple annotators performs the tracing and proofreading, with assistance of anatomy experts.^26^, ^27^, ^30^, ^31^ The exact segmentation approach depends on the complexity of the tissue and which type of annotation is desired (sparse or dense).

#### 5.2.2 Tracing, annotation and segmentation software

Annotation tools combine segmentation, annotation and visualisation into one interface. A distinction can be made between commercial and open‐source software. Examples of commercial software are Amira (Thermo Fisher Scientific), Imaris (Oxford Instruments) and Vision4D (Arivis), whereas often used open‐source programs are the Collaborative Annotation Toolkit for Massive Amounts of Image Data (CATMAID),^104^ KNOSSOS^105^ and its web version WebKnossos,^106^ Volume Annotation and Segmentation Tool (VAST)^107^ and earlier mentioned tools, TrakEM2,^100^ IMOD^101^ and MIB.^102^ Another distinction can be made between offline and web‐based tools. VAST, TrakEM2, IMOD and MIB are offline tools, while CATMAID and webKnossos retrieve image data and annotations hosted on a remote server and work with databases to manage annotations. It can be accessed anywhere (with an internet connection) and multiple annotators can simultaneously work on different parts of the volume. A comprehensive list comparing various features of all tools has been published elsewhere.^108^

### 5.3 Automated segmentation

The last 10 years have seen an increased usage and improvement of automated segmentation, made possible by developments in machine and deep learning. The choice for machine and/or deep learning seems obvious. Volume EM data sets are significantly growing in size, rendering complete manual segmentation impossible. Traditional segmentation methods most often fail or generalise poorly, because EM images are often noisy and characterised by variations in contrast and texture as well as artefacts introduced during sample preparation or imaging. Additionally, tissue structure can be very complex, such as the dense wiring patterns found in neural tissue. It has been shown that data driven models can cope with complex segmentation problems—convolutional neural networks (CNNs), from the domain of deep learning, outperformed traditional segmentation methods more than a decade ago.^109^ CNNs are very popular for image segmentation because they efficiently extract and combine information from different hierarchical levels in the image. Automated segmentation using deep learning has become the predominant strategy in two different domains: connectomics and cell biology.

#### 5.3.1 Automated segmentation in connectomics

In connectomics, the interest lies mainly in cell boundary segmentation and synapse detection. Many of the new developments can be attributed to several crowd‐sourcing competitions for automated segmentation in 2D and 3D: the International Symposium on Biomedical Imaging (ISBI),^110^ 3D segmentation of neurites in EM images (SNEMI3D)^i^ and circuit reconstruction from EM images (CREMI)^ii^ challenges. State‐of‐the‐art cell boundary segmentation approaches are typically either based on the popular U‐Net CNN architecture^111^, ^112^ or variants thereof. Variants based on U‐Net have achieved near‐human or even super‐human segmentation performance on neural EM data.^113^, ^114^, ^115^, ^116^, ^117^, ^118^ Alternatively, flood‐filling networks have been employed to increase segmentation accuracy at the expense of higher computational costs.^119^, ^120^ A comprehensive overview of these approaches and their implementation has been described elsewhere.^121^

Synaptic relations can be used to infer connectivity between neurons. Machine learning algorithms are therefore employed to find synaptic relations between neurons by classifying each voxel as ‘synaptic’ or ‘non‐synaptic’. Classical machine learning algorithms such as the random forest classifier are used,^122^, ^123^ but also here CNNs are gaining popularity.^124^, ^125^, ^126^, ^127^, ^128^, ^129^, ^130^, ^131^ In short, these methods try to predict candidate synapses and their directionality, while some also distinguish the pre‐ and postsynaptic neurons.^121^ Recent efforts in automated synapse detection resulted in a reliable connectivity graph in the whole brain fruit fly data set.^132^

#### 5.3.2 Cell organelle segmentation

In cell biology, the interest lies in segmentation of cell organelles to enable quantification of their morphology, distribution and size. A clear motive for this work is evidence that links alterations of organelle structure to neurodegenerative diseases and cancer.^4^, ^5^, ^6^, ^133^ The high axial resolution of SBF‐SEM and especially FIB‐SEM data allows for accurate segmentation of cell organelles. Due to the diversity of organelles and cell types as well as a lack of publicly available training data, automated organelle segmentation has not experienced the same surge as in connectomics, which has benefited from years of substantial manual annotation effort.^27^, ^30^, ^72^, ^134^, ^135^, ^136^ Nonetheless, there has been successful pioneering work within different types of volume EM data (Table 3). Similar to dense reconstructions of neural tissue, several studies have now demonstrated (fully) automated multiclass segmentation of organelles in single cells.^2^, ^3^

**TABLE 3:** Summary of studies on automatic organelle segmentation with machine learning and/or deep learning

Application	Organelle(s)	Technique	Network architecture	Reference
HeLa cells	NE	SBF‐SEM	U‐Net	^133^
HeLa cells, Jurkat cells, macrophages	Chromatin, ER, endosomal membranes, lysosome, MTs, Mito, NE, PM, vesicle membrane	FIB‐SEM	U‐Net	^2^
Rat hippocampus, mouse cortex	Mitochondria, ER	ATUM‐SEM, FIB‐SEM	ResNet, region proposal Net, Recursive Net, Mask R‐CNN	^5^
Rat hippocampus, mouse cortex	Mitochondria	ATUM‐SEM, FIB‐SEM	ResNet	^4^
Mouse primary beta cells	MTs, Golgi, centrioles, insulin granules	FIB‐SEM	U‐Net, random‐forest classifier	^3^
HeLa cells	ER, mitochondria, PM	FIB‐SEM	U‐Net, EfficientUnet	^186^
Mouse hippocampus	Vesicles, nuclei, mitochondria, membranes	SBF‐SEM	DeepEM3D	^154^
Human breast carcinoma	Nuclei, nucleoli	FIB‐SEM	ResNet, U‐Net	^46^
Mouse hippocampus	Mitochondria	FIB‐SEM	‘Conventional’ CNN	^139^
Mouse urinary bladder urothelial cells	Mitochondria, endolysosomes	FIB‐SEM	HighRes3DNet	^140^
Rat and human cortex	Mitochondria	ATUM‐MBSEM	U‐Net	^141^
Pancreatic beta cells	Insulin granules	FIB‐SEM	Multibranch FCN	^144^
Ovarian cancer cells	Extracellular vesicles	ssTEM	Fully residual U‐Net	^6^

*Note*: The organelles, EM technique and neural network architecture are indicated.

NE: nuclear envelope; ER: endoplasmic reticulum; MTs: microtubules; PM: plasma membrane.

Examples of important yet difficult segmentation problems in EM data include mitochondria, nuclei and vesicles. Mitochondria vary greatly in shape and size. This variation is not well represented in commonly used training data sets.^137^, ^138^ Nuclei segmentation is a common segmentation problem, also in light microscopy. Vesicles come in many forms and sizes. Automated mitochondria segmentation has been successfully applied to FIB‐SEM^4^, ^5^, ^139^, ^140^ and ATUM‐SEM data^4^, ^5^, ^141^ (despite its lower axial resolution). While it is possible to segment plasma and nuclear membranes with traditional segmentation algorithms,^142^, ^143^ two different groups approached nuclear envelope and nuclei segmentation with U‐Net variants.^46^, ^133^ To deal with the limited availability of expert manual annotations, the authors either aggregated multiple volunteer annotations^133^ or utilised sparse labelling techniques.^46^ Automated vesicle segmentation was developed for insulin granules^144^ and small extracellular vesicles.^6^

#### 5.3.3 Challenges with convolutional nets

There are several problems associated with CNN‐based segmentation. Generally, the performance is best on data sets with high isotropic resolution and proper alignment (SBF‐SEM, FIB‐SEM).^120^ Performance on serial section EM data, which is characterised by anisotropic resolution and slight defects in the alignment, can be improved by encouraging topologically correct segmentations obtained from the affinity graphs.^118^, ^145^ A second problem is that several methods do not generalise well outside of their particular source and tissues.^146^ To cope with this, domain adaption techniques can be used that transform the image content of different data sets to make them more similar to training data set.^147^, ^148^ On the other hand, training on data from various types of tissues may improve robustness.^138^ Lastly, problems arise due to artefacts introduced during sample preparation and imaging, which are rare in commonly used training data sets (e.g. CREMI, SMEMI3D). Solutions include increasing the occurrence artificially using data augmentation,^117^ locally realigning image sub volumes before region agglomeration^120^ or by supplementing these public data sets with manually segmented data from a portion of the imaging volume.

#### 5.3.4 Deep learning for the masses

Although automatic segmentation methods are becoming more powerful, they are often difficult to adopt by those with limited programming skills. To leverage the power of automatic segmentation in a more user‐friendly way, several state‐of‐the‐art algorithms and architectures have been integrated into popular image analysis tools. Fiji contains plugins such as ‘Trainable WEKA segmentation’ for interactive training of machine learning algorithms^149^ and ‘DeepImageJ^150^ for straightforward importing and deployment of deep learning models. Similarly, ilastik^151^ also supports simple and interactive training of machine learning algorithms and currently offers limited support for pre‐trained CNNs. Microscopy Image Browser (MIB) has been extended with a user‐friendly U‐Net.^152^ UNI‐EM^153^ is yet another user‐friendly tool that integrates multiple top‐performing 2D and 3D network architectures. Some tools work with cloud deployment to circumvent the need for local computational resources and software installation, such as DeepEM3D^154^ and ZeroCostDL4Mic.^155^

While these applications have reduced the barrier to entry for AI‐based analysis, there are several potential drawbacks. These include the limited number of implemented models and the verification of performance, as users generally look at visual segmentation quality without employing quantitative performance statistics. Furthermore, computational expertise and resources remain necessary for the documentation and maintenance of these tools. Lastly, different implementations require a varying level of knowledge about machine learning.

## 6 CHALLENGES IN DATA STORAGE, MANAGEMENT AND VISUALISATION

The output of volume EM is very information dense, but there are several hurdles in maximising its potential use. Currently, volume EM data set sizes range from several gigabytes to hundreds of terabytes, with several studies already having reached the petabyte scale.^29^, ^41^, ^45^ This has big implications for data storage and management. Data formats should be clear, accessible and complete to make sure data can be reused and revisited. Visualisation tools should offer fast terabyte scale data inspection. We will discuss the implications of the throughput increase and widened scope of volume EM methods on data management and visualisation.

### 6.1 Data storage

Where to store large volume EM data sets and their annotations? Small data sets can be managed on individual PCs or workstations, but with the current trend (Figure 1D) it is likely that storage on institutional servers or in the cloud will become the standard.

#### 6.1.1 Repositories and metadata

Systematic archiving of data and metadata in online repositories is not yet routine practice in the field of volume EM, though several dedicated repositories have emerged. These include the Electron Microscopy Public Image Archive (EMPIAR),^156^ Image Data Resource (IDR)^157^ and the EMBL BioImage Archiveiii (BIA). EMPIAR is EM specific, whereas IDR and BIA are more broad. Currently, these repositories allow the download and upload of whole data sets, but it may be easier to interact with (a subset of) the data via application programming interfaces (APIs) or viewers. How data should be formatted and stored is an ongoing discussion in the EM community. While it is generally accepted that EM data should follow the FAIR format^158^ to maximise reuse, it is difficult to standardise metadata because the needs vary greatly based on the application or imaging modality. Nonetheless, recently a set of guidelines for Recommended Metadata for Biological Images (REMBI) was published.^159^ This will be incorporated as a standard for submission into IDR.

#### 6.1.2 Data formats

How is volume EM data stored? Different file formats are used depending on the application and storage location. During acquisition, data are often saved in proprietary microscopy data formats, which are optimised for writing. For visualisation purposes, however, the optimal format is entirely different. Data with high lateral but low axial resolution (i.e. ssTEM, ATUM‐SEM) are made possible by making use of pyramids of increasingly downsampled flat images (*tiles*), either remotely (CATMAID) or locally (TrakEM2). This is convenient because these images are usually viewed in 2D. Data with high axial resolution (FIB‐SEM, SBF‐SEM) is instead saved in a cube format (employed by KNOSSOS) which makes browsing or reslicing in the *z* direction faster and easier. In data archiving, flexible file extensions such as TIFF and HDF5 are used, which can store multidimensional pyramidal data with associated metadata. However, data from TIFFs can only be read as individual 2D tiles, while HDF5 and other ‘next‐generation file formats’ such as N5^iv^ and Zarr^v^ allow reading and writing of three‐dimensional chunks of images to separate, smaller files, which is much faster and better suited for cloud storage.^160^

### 6.2 Data management

**FIGURE 6:**
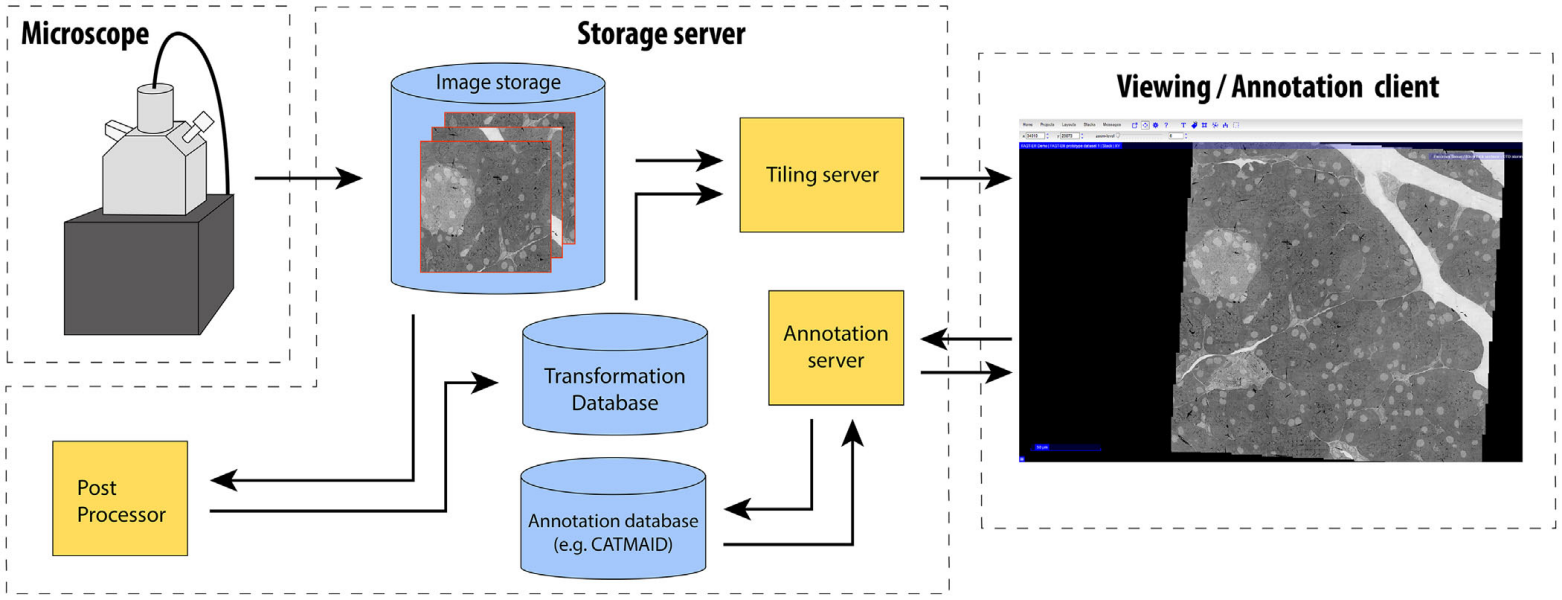
Example of data management structure. The image data are acquired by an electron microscope and stored within a central database. The database is connected to a post‐processing service which performs image processing and computes the transformations between the images, which are contained in a separate database. The annotations are contained in a separate database (here, CATMAID^104^). A client can send requests to the image, transformation and annotation database to view certain image data and corresponding annotations. The image data is tiled before it is sent. The arrows indicate the flow of information.

Client‐server applications are becoming a popular tool to interact with volume EM data.^49^, ^104^, ^106^, ^161^ Plugins for processing, visualisation and annotation can be remotely installed, and there is no need to download data or install software locally other than a web browser. Moreover, research data can be more easily shared as data can be made accessible to multiple users from different locations simultaneously. The data and metadata are stored in remote servers, while the user retrieves the data via a client (Figure 6).

#### 6.2.1 ‘Local’ data management

An open, flexible and scalable data management platform suitable for electron microscopy data is Open Microscopy Environment Remote Objects (OMERO).^162^ It was created with the idea of standardising data access. Data can be imported using Bio‐Formats, which converts proprietary microscopy data formats into a common data model (currently, OME XML with OME‐TIFF).^163^

#### 6.2.2 Cloud data management

Apart from the costs, hosting EM data in the cloud offers several advantages in terms of convenience and accessibility. One such example is Neurodata.io,^vi^ ^164^ a community‐developed and maintained software ecosystem for neuroscience data deployed in the commercial cloud (AWS), containing TrakEM2 for registration, NeuroGlancervii for visualisation and BossDB^165^ for data management. Multiple data sets of different formats and imaging modalities can be combined. In addition, the hosted data can be accessed via annotation software (VAST/KNOSSOS). Another example is OpenOrganelle, a repository for cell biology data, created at the Janelia Research Campus and also hosted in the cloud.^166^ The platform hosts 10 FIB‐SEM data sets of various cell lines and tissues for online visualisation and offline data mining, while at the same time providing the code and tutorials for all available tools. Separately, the entire EM volume of *Platynereis dumerilii* is hosted in N5 format in cloud object storage at EMBL and can be accessed using an N5 API. The data can be browsed using a specifically designed Fiji plugin ‘PlatyBrowser’ in MoBIE.^49^ These projects demonstrate the potential and conveniences of cloud data management of volume EM data.

### 6.3 Developing scalable architectures

Apart from developing new tools, there is the challenge to make scalable architectures out of existing tools. Employing these tools on high performance computing (HPC) clusters allows large data sets to be processed in parallel. Vescovi et al.^167^ developed a scalable and modular pipeline which integrates multiple software modalities such as TrakEM2, NeuroGlancer and Flood‐Filling networks to perform several tasks from registration to annotation and visualisation. These tools are made HPC deployable and wrapped in an operational database which can be used to create custom pipelines for image processing and annotation.

### 6.4 Terabyte data viewers

Some annotation tools have been designed with large data sets in mind. Examples are VAST^107^ and NeuTu.^161^ VAST is mainly a segmentation and annotation tool, and is able to handle very large data sets, which can be imported from a server or locally. Manual segmentation at different zoom levels is also supported, although simultaneous editing by multiple users is not. In contrast, NeuTu allows collective proofreading and correcting errors created by automated segmentation by manually merging or splitting segments. It is part of DVID,^168^ a distributed, versioned, image‐orientated data service in which NeuTu acts as the data client. DVID works with 2D and 3D data and has a version control feature to manage different annotation states. Lastly, BigDataViewer^169^ and Multimodal Big Image Data Exploration (MoBIE),^49^ both Fiji plugins, make use of the convenient HDF5 format to interactively navigate and visualise large image sequences. MoBIE is additionally equipped with an object storage backend to load data from remote sources.

### 6.5 Collective annotation

The last 10 years have seen the emergence of projects in which researchers can collectively work on annotating volume EM data. Some of these endeavours actively encourage non‐scientists to participate through ‘citizen science’ initiatives. Non‐scientists can help with proofreading annotations produced by automated segmentation algorithms (for example in the game Eyewire^135^) or they can assist in generating training data for deep learning applications (e.g. ‘Etch a Cell^133^). The fruit fly community has developed FlyWire,^136^ with the goal of collectively mapping the fruit fly connectome from whole brain data sets.^27^, ^28^ It resembles EyeWire but is currently limited to researchers only.

## 7 CONCLUSION AND OUTLOOK

Almost 10 years after the review of Briggman and Bock,^1^ there has not yet emerged a volume EM technique that makes others obsolete. FIB‐SEM and to some extent SBF‐SEM remain the methods of choice in studies where high isotropic resolution is favoured over throughput, for example in cell biology. The high isotropic resolution also provides the advantage of more precise automated segmentation. ‘Enhanced FIB‐SEM’ allows the imaging of larger samples. The implementation of new milling approaches will hopefully speed up FIB‐SEM even further. ssTEM has seen several innovations (TEMCA, GridTape, AutoTEM) that greatly improve the throughput. It offers the highest lateral resolution, but its limited axial resolution and artefacts hamper automated image analysis. For all techniques, a considerable amount of manual proofreading remains necessary after automated segmentation. This results in many studies still heavily relying on manual annotation efforts. Nevertheless, the performance of automated segmentation algorithms will likely improve further given their recent introduction in the field and the general interest in AI research.

Multibeam SEM should not be ignored. The possibility to increase the throughput of a single microscope by orders of magnitude is very cost‐effective. However, both approaches are (for the moment) incompatible with block‐face approaches, making them dependent on serial sectioning. Nevertheless, the speed of MB‐SEM could be key in rapid diagnostics (digital pathology)^170^ but also in studying brain development in multiple specimens.^40^, ^171^, ^172^ The MultiSEM has demonstrated compatibility with ATUM. FAST‐EM still has to demonstrate compatibility with a high‐throughput sectioning approach.

Currently, the expertise in large volume EM is limited to several research groups. Centralised imaging facilities could take a leading role in investing in high‐throughput electron microscopes and the elaborate data infrastructure required for these machines. Will it be possible to image and annotate a full adult zebrafish in the near future, maybe even a mouse brain? Will the imaging and annotation of a small animal brain follow a path similar to, for example, the human genome project? An average adult mouse brain has a volume of 485–530 mm^3^.^173^ It is clear that more automation is needed. Advancements in methodology will probably again play a key role, but it will also require extensive collaboration and sharing of resources.

Information from high‐throughput studies, such as connectomes of animals or atlases of healthy and diseased tissue, will presumably give critical insights. It can lead to direct discoveries or provide a starting point to test hypotheses on the relation between structural changes and disease onset with functional studies. Novel data mining approaches and meta‐analyses of various data sets could give new insights in ultrastructural differences between data sets of various tissues and animals, similar to microarray studies.

In conclusion, methodological improvements are making volume EM more accessible and are alleviating the burden on throughput. Automated segmentation methods are reducing the workload of manual annotation, but considerable human effort remains necessary. In the field of data management, there is a need for a joint approach on how to manage large volume EM data. The adoption of a common file format could improve collaboration and simplify training of automated segmentation methods. We see an opportunity for open hosting of data sets with corresponding annotations to maximise the profit to the community. The potential of volume EM may be greater than ever before.

## AUTHOR CONTRIBUTIONS

ECC initiated the project and suggested the topic. AJK conducted the literature survey, drafted the original manuscript and produced the figures. RL revised the manuscript and contributed to Sections 4, 5 and 6. JPH and ECC supervised the writing process and offered critical remarks on the manuscript and figures.

## FUNDING

AJK and JPH acknowledge support from the ECSEL Joint Under‐taking (JU) under grant agreement No. 826589. The JU receives support from the European Union's Horizon 2020 research and innovation programme and Netherlands, Belgium, Germany, France, Italy, Austria, Hungary, Romania, Sweden and Israel. RL, ECC, JPH further acknowledge support from the EU‐React G4P4 ‘Kansen voor West’ project IMDAP.

## COMPETING INTERESTS

AJK, RL and ECC declare no competing interests. JPH is co‐founder of and shareholder in Delmic BV, a company selling integrated microscopes, including FAST‐EM.

## Supporting information

Supplementary Material

## Footnotes

^i^

http://brainiac2.mit.edu/SNEMI3D/home.

^ii^

https://cremi.org/.

^iii^

https://www.ebi.ac.uk/bioimage‐archive/.

^iv^

https://github.com/saalfeldlab/n5.

^v^

https://github.com/zarr‐developers.

^vi^

https://neurodata.io/.

^vii^

https://github.com/google/neuroglancer.

## References

[1] Briggman, K. L. , & Bock, D. D. (2012). Volume electron microscopy for neuronal circuit reconstruction. Current Opinion in Neurobiology, 22(1), 154–161. 22119321 10.1016/j.conb.2011.10.022

[2] Heinrich, L. , Bennett, D. , Ackerman, D. , Park, W. , Bogovic, J. , Eckstein, N. , … COSEM Project Team . (2021). Whole‐cell organelle segmentation in volume electron microscopy. Nature, 599, 141–146. 34616042 10.1038/s41586-021-03977-3

[3] Müller, A. , Schmidt, D. , Xu, C. S. , Pang, S. , D'Costa, J. V. , Kretschmar, S. , & Solimena, M. (2020). 3D FIB‐SEM reconstruction of microtubule–organelle interaction in whole primary mouse β cells. Journal of Cell Biology, *220*(2). https://rupress.org/jcb/article/220/2/e202010039/211599/3D-FIB-SEM-reconstruction-of-microtubule-organelle 10.1083/jcb.202010039 PMC7748794 33326005

[4] Xiao, C. , Chen, X. , Li, W. , Li, L. , Wang, L. , Xie, Q. , & Han, H. (2018). Automatic mitochondria segmentation for EM data using a 3D supervised convolutional network. Frontiers in Neuroanatomy, 12, 92. 30450040 10.3389/fnana.2018.00092 PMC6224513

[5] Liu, J. , Li, L. , Yang, Y. , Hong, B. , Chen, X. , Xie, Q. , & Han, H. (2020). Automatic reconstruction of mitochondria and endoplasmic reticulum in electron microscopy volumes by deep learning. Frontiers in Neuroscience, *14*. https://www.frontiersin.org/articles/10.3389/fnins.2020.00599/full 10.3389/fnins.2020.00599 PMC7394701 32792893

[6] Gómez‐de‐Mariscal, E. , Maška, M. , Kotrbová, A. , Pospíchalová, V. , Matula, P. , & Muñoz‐Barrutia, A. (2019). Deep‐learning‐based segmentation of small extracellular vesicles in transmission electron microscopy images. Scientific Reports, 9(1), 1–10. 31519998 10.1038/s41598-019-49431-3 PMC6744556

[7] Harris, J. R. (2015). Transmission electron microscopy in molecular structural biology: A historical survey. Archives of Biochemistry and Biophysics, 581, 3–18. 25475529 10.1016/j.abb.2014.11.011

[8] Birch‐Andersen, A. (1955). Reconstruction of the nuclear sites of *Salmonella typhimurium* from electron microǵraphs of serial sections. Microbiology (Reading, England), 13(2), 327–329. 10.1099/00221287-13-2-327 13278481

[9] Bang, B. G. , & Bang, F. B. (1957). Graphic reconstruction of the third dimension from serial electron microphotographs. Journal of Ultrastructure Research, 1(2), 138–46. 13492346 10.1016/s0022-5320(57)80002-1

[10] Stevens, J. K. , Davis, T. L. , Friedman, N. , & Sterling, P. (1980). A systematic approach to reconstructing microcircuitry by electron microscopy of serial sections. Brain Research Reviews, 2(1–3), 265–93. 10.1016/0165-0173(80)90010-7 6258704

[11] Harris, K. M. , Perry, E. , Bourne, J. , Feinberg, M. , Ostroff, L. , & Hurlburt, J. (2006). Uniform serial sectioning for transmission electron microscopy. Journal of Neuroscience, 26(47), 12101–3. 17122034 10.1523/JNEUROSCI.3994-06.2006 PMC6675417

[12] Peddie, C. J. , & Collinson, L. M. (2014). Exploring the third dimension: Volume electron microscopy comes of age. Micron (Oxford, England: 1993), 61, 9–19. 10.1016/j.micron.2014.01.009 24792442

[13] Soto, G. E. , Young, S. J. , Martone, M. E. , Deerinck, T. J. , Lamont, S. , & Carragher, B. O. (1994). Serial section electron tomography: A method for three‐dimensional reconstruction of large structures. Neuroimage, 1(3), 230–43. 9343574 10.1006/nimg.1994.1008

[14] Bárcena, M. , & Koster, A. J. (2009). Electron tomography in life science. Seminars in Cell & Developmental Biology, 20, 920–30. 19664718 10.1016/j.semcdb.2009.07.008 PMC7110493

[15] Hohmann‐Marriott, M. F. , Sousa, A. A. , Azari, A. A. , Glushakova, S. , Zhang, G. , & Zimmerberg, J. (2009). Nanoscale 3D cellular imaging by axial scanning transmission electron tomography. Nature Methods, 6(10), 729–31. 19718033 10.1038/nmeth.1367 PMC2755602

[16] Denk, W. , & Horstmann, H. (2004). Serial block‐face scanning electron microscopy to reconstruct three‐dimensional tissue nanostructure. PLoS Biology, 2(11), e329. 15514700 10.1371/journal.pbio.0020329 PMC524270

[17] Heymann, J. A. W. , Hayles, M. , Gestmann, I. , Giannuzzi, L. A. , Lich, B. , & Subramaniam, S. (2006). Site‐specific 3D imaging of cells and tissues with a dual beam microscope. Journal of Structural Biology, 155(1), 63–73. 16713294 10.1016/j.jsb.2006.03.006 PMC1647295

[18] Knott, G. , Marchman, H. , Wall, D. , & Lich, B. (2008). Serial section scanning electron microscopy of adult brain tissue using focused ion beam milling. Journal of Neuroscience, 28(12), 2959–64. 18353998 10.1523/JNEUROSCI.3189-07.2008 PMC6670719

[19] Micheva, K. D. , & Smith, S. J. (2007). Array tomography: A new tool for imaging the molecular architecture and ultrastructure of neural circuits. Neuron, 55(1), 25–36. 17610815 10.1016/j.neuron.2007.06.014 PMC2080672

[20] Wacker, I. , & Schroeder, R. R. (2013). Array tomography. Journal of Microscopy, 252(2), 93–9. 24111814 10.1111/jmi.12087

[21] Hayworth, K. J. , Kasthuri, N. , Schalek, R. , & Lichtman, J. W. (2006). Automating the collection of ultrathin serial sections for large volume TEM reconstructions. Microscopy and Microanalysis, 12(S02), 86–7.

[22] Schalek, R. , Wilson, A. , Lichtman, J. , Josh, M. , Kasthuri, N. , & Berger, D. (2012). ATUM‐based SEM for high‐speed large‐volume biological reconstructions. Microscopy and Microanalysis, 18(S2), 572–3.

[23] Baena, V. , Conrad, R. , Friday, P. , Fitzgerald, E. , Kim, T. , & Bernbaum, J. (2021). FIB‐SEM as a volume electron microscopy approach to study cellular architectures in SARS‐CoV‐2 and other viral infections: A practical primer for a virologist. Viruses, 13(4), 611. 33918371 10.3390/v13040611 PMC8066521

[24] Wolff, G. , & Bárcena, M. (2021). Multiscale electron microscopy for the study of viral replication organelles. Viruses, 13(2), 197. 33525547 10.3390/v13020197 PMC7912242

[25] Narayan, K. , & Subramaniam, S. (2015). Focused ion beams in biology. Nature Methods, 12(11), 1021–31. 26513553 10.1038/nmeth.3623 PMC6993138

[26] Hildebrand, D. G. C. , Cicconet, M. , Torres, R. M. , Choi, W. , Quan, T. M. , & Moon, J. (2017). Whole‐brain serial‐section electron microscopy in larval zebrafish. Nature, 545(7654), 345–349. 28489821 10.1038/nature22356 PMC5594570

[27] Zheng, Z. , Lauritzen, J. S. , Perlman, E. , Robinson, C. G. , Nichols, M. , & Milkie, D. (2018). A complete electron microscopy volume of the brain of adult *Drosophila melanogaster* . Cell, 174(3), 730–43. 30033368 10.1016/j.cell.2018.06.019 PMC6063995

[28] Scheffer, L. K. , Xu, C. S. , Januszewski, M. , Lu, Z. , Takemura, S.‐Y. , & Hayworth, K. J. (2020). A connectome and analysis of the adult Drosophila central bra. *BioRxiv*. 10.7554/eLife.57443 PMC7546738 32880371

[29] Yin, W. , Brittain, D. , Borseth, J. , Scott, M. E. , Williams, D. , & Perkins, J. (2020). A petascale automated imaging pipeline for mapping neuronal circuits with high‐throughput transmission electron microscopy. Nature Communications, 11(1), 1–12. 10.1038/s41467-020-18659-3 PMC7532165 33009388

[30] Helmstaedter, M. , Briggman, K. L. , Turaga, S. C. , Jain, V. , Seung, H. S. , & Denk, W. (2013). Connectomic reconstruction of the inner plexiform layer in the mouse retina. Nature, 500(7461), 168–74. 23925239 10.1038/nature12346

[31] Motta, A. , Berning, M. , Boergens, K. M. , Staffler, B. , Beining, M. , & Loomba, S. (2019). Dense connectomic reconstruction in layer 4 of the somatosensory cortex. Science, *366*(6469). https://www.science.org/doi/10.1126/science.aay3134 10.1126/science.aay3134 31649140

[32] Eberle, A. L. , Mikula, S. , Schalek, R. , Lichtman, J. , Tate, M. L. K. , & Zeidler, D. (2015). High‐resolution, high‐throughput imaging with a multibeam scanning electron microscope. Journal of Microscopy, 259(2), 114–20. 25627873 10.1111/jmi.12224 PMC4670696

[33] Ren, Y. , & Kruit, P. (2016). Transmission electron imaging in the Delft multibeam scanning electron microscope 1. Journal of Vacuum Science & Technology B, Nanotechnology and Microelectronics: Materials, Processing, Measurement, and Phenomena, 34(6), 06KF02.

[34] Hayworth, K. J. , Xu, C. S. , Lu, Z. , Knott, G. W. , Fetter, R. D. , & Tapia, J. C. (2015). Ultrastructurally smooth thick partitioning and volume stitching for large‐scale connectomics. Nature Methods, 12(4), 319–22. 25686390 10.1038/nmeth.3292 PMC4382383

[35] Bock, D. D. , Lee, W.‐C. A. , Kerlin, A. M. , Andermann, M. L. , Hood, G. , & Wetzel, A. W. (2011). Network anatomy and in vivo physiology of visual cortical neurons. Nature, 471(7337), 177–82. 21390124 10.1038/nature09802 PMC3095821

[36] Xu, C. S. , Hayworth, K. J. , Lu, Z. , Grob, P. , Hassan, A. M. , & Garcia‐Cerdan, J. G. (2017). Enhanced FIB‐SEM systems for large‐volume 3D imaging. Elife, 6, e25916. 28500755 10.7554/eLife.25916 PMC5476429

[37] Phelps, J. S. , Hildebrand, D. G. C. , Graham, B. J. , Kuan, A. T. , Thomas, L. A. , & Nguyen, T. M. (2021). Reconstruction of motor control circuits in adult Drosophila using automated transmission electron microscopy. Cell, 184(3), 759–74. 33400916 10.1016/j.cell.2020.12.013 PMC8312698

[38] Kremer, A. , Lippens, S. , Bartunkova, S. , Asselbergh, B. , Blanpain, C. , & Fendrych, M. (2015). Developing 3D SEM in a broad biological context. Journal of Microscopy, 259(2), 80–96. 25623622 10.1111/jmi.12211 PMC4670703

[39] Kornfeld, J. , & Denk, W. (2018). Progress and remaining challenges in high‐throughput volume electron microscopy. Current Opinion in Neurobiology, 50, 261–7. 29758457 10.1016/j.conb.2018.04.030

[40] Witvliet, D. , Mulcahy, B. , Mitchell, J. K. , Meirovitch, Y. , Berger, D. R. , & Wu, Y. (2021). Connectomes across development reveal principles of brain maturation. Nature, 596(7871), 257–61. 34349261 10.1038/s41586-021-03778-8 PMC8756380

[41] Shapson‐Coe, A. , Januszewski, M. , Berger, D. R. , Pope, A. , Wu, Y. , & Blakely, T. (2021). A connectomic study of a petascale fragment of human cerebral cortex. *BioRxiv*.

[42] Pinali, C. , Bennett, H. , Davenport, J. B. , Trafford, A. W. , & Kitmitto, A. (2013). Three‐dimensional reconstruction of cardiac sarcoplasmic reticulum reveals a continuous network linking transverse‐tubules: This organization is perturbed in heart failure. Circulation Research, 113(11), 1219–1230. 24044951 10.1161/CIRCRESAHA.113.301348

[43] Xu, C. S. , Pang, S. , Hayworth, K. J. , & Hess, H. F. (2020). Transforming FIB‐SEM systems for large‐volume connectomics and cell biology. In: I. Wacker , E. Hummel , S. Burgold , & R. Schröder , (Eds.), Volume microscopy. Neuromethods (vol. 155, p. 221–243). New York, NY: Humana.

[44] Schneider, J. P. , Hegermann, J. , & Wrede, C. (2020). Volume electron microscopy: Analyzing the lung. Histochemistry and Cell Biology, 155, 241–260. 32944795 10.1007/s00418-020-01916-3 PMC7910248

[45] Bae, J. A. , Baptiste, M. , Bodor, A. L. , Brittain, D. , Buchanan, J. A. , & Bumbarger, D. J. (2021). Functional connectomics spanning multiple areas of mouse visual cortex. *BioRxiv*. 10.1038/s41586-025-08790-w PMC11981939 40205214

[46] Machireddy, A. , Thibault, G. , Loftis, K. G. , Stoltz, K. , Bueno, C. E. , & Smith, H. R. (2021) Robust segmentation of cellular ultrastructure on sparsely labeled 3D electron microscopy images using deep learning. Available at SSRN 3830021. 10.3389/fbinf.2023.1308708 PMC10754953 38162124

[47] Wei, D. , Jacobs, S. , Modla, S. , Zhang, S. , Young, C. L. , & Cirino, R. (2012). High‐resolution three‐dimensional reconstruction of a whole yeast cell using focused‐ion beam scanning electron microscopy. Biotechniques, 53(1), 41–8. 22780318 10.2144/000113850

[48] Hughes, L. , Borrett, S. , Towers, K. , Starborg, T. , & Vaughan, S. (2017). Patterns of organelle ontogeny through a cell cycle revealed by whole‐cell reconstructions using 3D electron microscopy. Journal of Cell Science, 130(3), 637–47. 28049718 10.1242/jcs.198887

[49] Vergara, H. M. , Pape, C. , Meechan, K. I. , Zinchenko, V. , Genoud, C. , & Wanner, A. A. (2021). Whole‐body integration of gene expression and single‐cell morphology. Cell, 184(18), 4819–37. 34380046 10.1016/j.cell.2021.07.017 PMC8445025

[50] Armer, H. E. J. , Mariggi, G. , Png, K. M. Y. , Genoud, C. , Monteith, A. G. , & Bushby, A. J. (2009). Imaging transient blood vessel fusion events in zebrafish by correlative volume electron microscopy. PLoS One, 4(11), e7716. 19893745 10.1371/journal.pone.0007716 PMC2769265

[51] Svensson, R. B. , Herchenhan, A. , Starborg, T. , Larsen, M. , Kadler, K. E. , & Qvortrup, K. (2017). Evidence of structurally continuous collagen fibrils in tendons. Acta Biomaterialia, 50, 293–301. 28063986 10.1016/j.actbio.2017.01.006

[52] Parlakgul, G. , Arruda, A. P. , Pang, S. , Cagampan, E. , Min, N. , & Hotamışlıgil, G. S. (2022). Regulation of liver subcellular architecture controls metabolic homeostasis. Nature, 603, 736–742. 35264794 10.1038/s41586-022-04488-5 PMC9014868

[53] Felts, R. L. , Narayan, K. , Estes, J. D. , Shi, D. , Trubey, C. M. , & Fu, J. (2010). 3D visualization of HIV transfer at the virological synapse between dendritic cells and T cells. Proceedings of the National Academy of Sciences, 107(30), 13336–41. 10.1073/pnas.1003040107 PMC2922156 20624966

[54] Vihinen, H. , Belevich, I. , & Jokitalo, E. (2013). Three dimensional electron microscopy of cellular organelles by serial block face SEM and ET. Microscopy and Analysis, 27, 7–10.

[55] Hoffman, D. P. , Shtengel, G. , Xu, C. S. , Campbell, K. R. , Freeman, M. , & Wang, L. (2020). Correlative three‐dimensional super‐resolution and block‐face electron microscopy of whole vitreously frozen cells. Science, *367*(6475). https://www.science.org/doi/10.1126/science.aaz5357?url_ver=Z39.88-2003&rfr_id=ori:rid:crossref.org&rfr_dat=cr_pub%20%200pubmed 10.1126/science.aaz5357 PMC7339343 31949053

[56] Cretoiu, D. , Hummel, E. , Zimmermann, H. , Gherghiceanu, M. , & Popescu, L. M. (2014). Human cardiac telocytes: 3D imaging by FIB‐SEM tomography. Journal of Cellular and Molecular Medicine, 18(11), 2157–64. 25327290 10.1111/jcmm.12468 PMC4224550

[57] Schneider, J. P. , Wrede, C. , & Mühlfeld, C. (2020). The three‐dimensional ultrastructure of the human alveolar epithelium revealed by focused ion beam electron microscopy. International Journal of Molecular Sciences, 21(3), 1089. 10.3390/ijms21031089 PMC7038159 32041332

[58] Mohammadi‐Gheidari, A. , Hagen, C. W. , & Kruit, P. (2010). Multibeam scanning electron microscope: Experimental results. Journal of Vacuum Science & Technology B, Nanotechnology and Microelectronics: Materials, Processing, Measurement, and Phenomena, 28(6), C6G5–C6G10.

[59] Meisburger, D. , Spallas, J. , Werder, K. , & Muray, L. (2015). Proposed architecture of a multicolumn electron‐beam wafer inspection system for high‐volume manufacturing. Journal of Vacuum Science & Technology B, Nanotechnology and Microelectronics: Materials, Processing, Measurement, and Phenomena, 33(6), 06FN01.

[60] Kemen, T. , Malloy, M. , Thiel, B. , Mikula, S. , Denk, W. , & Dellemann, G. (2015). Further advancing the throughput of a multi‐beam SEM. Metrology, Inspection, and Process Control for Microlithography, *XXIX*, International Society for Optics and Photonics, vol. 9424, p. 94241U.

[61] Riedesel, C. , Müller, I. , Kaufmann, N. , Adolf, A. , Kämmer, N. , & Fritz, H. (2019). First demonstration of a 331‐beam SEM. Metrology, Inspection, and Process Control for Microlithography, *XXXIII*, International Society for Optics and Photonics, vol. 10959, p. 1095931.

[62] Kruit, P. , & Zuidema, W. (2019). A dedicated multi‐beam SEM for transmission imaging of thin samples. Microscopy and Microanalysis, 25(S2), 1034–5.

[63] Shibata, S. , Iseda, T. , Mitsuhashi, T. , Oka, A. , Shindo, T. , & Moritoki, N. (2019). Large‐area fluorescence and electron microscopic correlative imaging with multibeam scanning electron microscopy. Frontiers in Neural Circuits, 13, 29. 31133819 10.3389/fncir.2019.00029 PMC6517476

[64] Günther, A. , Dedek, K. , Haverkamp, S. , Irsen, S. , Briggman, K. L. , & Mouritsen, H. (2021). Double cones and the diverse connectivity of photoreceptors and bipolar cells in an avian retina. Journal of Neuroscience, 41(23), 5015–28. 33893221 10.1523/JNEUROSCI.2495-20.2021 PMC8197639

[65] Lee, W.‐C. A. , Bonin, V. , Reed, M. , Graham, B. J. , Hood, G. , & Glattfelder, K. (2016). Anatomy and function of an excitatory network in the visual cortex. Nature, 532(7599), 370–4. 27018655 10.1038/nature17192 PMC4844839

[66] Graham, B. J. , Hildebrand, D. G. C. , Kuan, A. T. , Maniates‐Selvin, J. T. , Thomas, L. A. , & Shanny, B. L. (2019). High‐throughput transmission electron microscopy with automated serial sectioning. bioRxiv, 657346.

[67] Hayworth, K. J. , Peale, D. , Januszewski, M. , Knott, G. W. , Lu, Z. , & Xu, C. S. (2020). Gas cluster ion beam SEM for imaging of large tissue samples with 10 nm isotropic resolution. Nature Methods, 17(1), 68–71. 31740820 10.1038/s41592-019-0641-2

[68] Winiarski, B. , Gholinia, A. , Mingard, K. , Gee, M. , Thompson, G. E. , & Withers, P. J. (2017). Broad ion beam serial section tomography. Ultramicroscopy, 172, 52–64. 27863288 10.1016/j.ultramic.2016.10.014

[69] Burnett, T. L. , Kelley, R. , Winiarski, B. , Contreras, L. , Daly, M. , & Gholinia, A. (2016). Large volume serial section tomography by Xe Plasma FIB dual beam microscopy. Ultramicroscopy, 161, 119–29. 26683814 10.1016/j.ultramic.2015.11.001

[70] Gholinia, A. , Curd, M. , Bousser, E. , Taylor, K. , Hosman, T. , & Coyle, S. (2020). Coupled broad ion beam‐scanning electron microscopy (BIB‐SEM) for polishing and three dimensional (3D) serial section tomography (SST). Ultramicroscopy, 214, 112989. 10.1016/j.ultramic.2020.112989 32416435

[71] Sergey, G. , Denis, K. , Ava, H. , Gediminas, G. , Viola, O. , & HP, L. R. (2018). Oxygen plasma focused ion beam scanning electron microscopy for biological samples. bioRxiv, 457820.

[72] Kasthuri, N. , Hayworth, K. J. , Berger, D. R. , Schalek, R. L. , Conchello, J. A. , & Knowles‐Barley, S. (2015). Saturated reconstruction of a volume of neocortex. Cell, 162(3), 648–61. 26232230 10.1016/j.cell.2015.06.054

[73] Morgan, J. L. , Berger, D. R. , Wetzel, A. W. , & Lichtman, J. W. (2016). The fuzzy logic of network connectivity in mouse visual thalamus. Cell, 165(1), 192–206. 27015312 10.1016/j.cell.2016.02.033 PMC4808248

[74] Kislinger, G. , Gnägi, H. , Kerschensteiner, M. , Simons, M. , Misgeld, T. , & Schifferer, M. (2020). Multiscale ATUM‐FIB microscopy enables targeted ultrastructural analysis at isotropic resolution. Iscience, 23(7), 101290. 32622266 10.1016/j.isci.2020.101290 PMC7334410

[75] Tobin, W. F. , Wilson, R. I. , & Lee, W.‐C. A. (2017). Wiring variations that enable and constrain neural computation in a sensory microcircuit. Elife, 6, e24838. 28530904 10.7554/eLife.24838 PMC5440167

[76] Schmidt, H. , Gour, A. , Straehle, J. , Boergens, K. M. , Brecht, M. , & Helmstaedter, M. (2017). Axonal synapse sorting in medial entorhinal cortex. Nature, 549(7673), 469–75. 28959971 10.1038/nature24005

[77] Seligman, A. M. , Wasserkrug, H. L. , & Hanker, J. S. (1966). A new staining method (OTO) for enhancing contrast of lipid‐containing membranes and droplets in osmium tetroxide‐fixed tissue with osmiophilic thiocarbohydrazide (TCH). The Journal of Cell Biology, 30(2), 424. 4165523 10.1083/jcb.30.2.424 PMC2106998

[78] Tapia, J. C. , Kasthuri, N. , Hayworth, K. J. , Schalek, R. , Lichtman, J. W. , & Smith, S. J. (2012). High‐contrast en bloc staining of neuronal tissue for field emission scanning electron microscopy. Nature Protocols, 7(2), 193. 22240582 10.1038/nprot.2011.439 PMC3701260

[79] Hua, Y. , Laserstein, P. , & Helmstaedter, M. (2015). Large‐volume en‐bloc staining for electron microscopy‐based connectomics. Nature Communications, 6(1), 1–7. 10.1038/ncomms8923 PMC4532871 26235643

[80] Mikula, S. , & Denk, W. (2015). High‐resolution whole‐brain staining for electron microscopic circuit reconstruction. Nature Methods, 12(6), 541–6. 25867849 10.1038/nmeth.3361

[81] Genoud, C. , Titze, B. , Graff‐Meyer, A. , & Friedrich, R. W. (2018). Fast homogeneous en bloc staining of large tissue samples for volume electron microscopy. Frontiers in Neuroanatomy, 12, 76. 30323746 10.3389/fnana.2018.00076 PMC6172304

[82] Wacker, I. , Spomer, W. , Hofmann, A. , Thaler, M. , Hillmer, S. , & Gengenbach, U. (2016). Hierarchical imaging: A new concept for targeted imaging of large volumes from cells to tissues. BMC Cell Biology, 17(1), 1–12. 27955619 10.1186/s12860-016-0122-8 PMC5154069

[83] Koike, T. , Kataoka, Y. , Maeda, M. , Hasebe, Y. , Yamaguchi, Y. , & Suga, M. (2017). A device for ribbon collection for array tomography with scanning electron microscopy. Acta Histochemica Et Cytochemica, 50(5), 135–40. 29276315 10.1267/ahc.17013 PMC5736830

[84] Lee, T. J. , Kumar, A. , Balwani, A. H. , Brittain, D. , Kinn, S. , & Tovey, C. A. (2018). Large‐scale neuroanatomy using LASSO: Loop‐based automated serial sectioning operation. PLoS One, 13(10), e0206172. 30352088 10.1371/journal.pone.0206172 PMC6198950

[85] Kubota, Y. , Sohn, J. , Hatada, S. , Schurr, M. , Straehle, J. , & Gour, A. (2018). A carbon nanotube tape for serial‐section electron microscopy of brain ultrastructure. Nature Communications, 9(1), 1–15. 10.1038/s41467-017-02768-7 PMC5789869 29382816

[86] Templier, T. (2019). MagC, magnetic collection of ultrathin sections for volumetric correlative light and electron microscopy. Elife, 8, e45696. 31294691 10.7554/eLife.45696 PMC6697447

[87] Deerinck, T. J. , Shone, T. M. , Bushong, E. A. , Ramachandra, R. , Peltier, S. T. , & Ellisman, M. H. (2018). High‐performance serial block‐face SEM of nonconductive biological samples enabled by focal gas injection‐based charge compensation. Journal of Microscopy, 270(2), 142–9. 29194648 10.1111/jmi.12667 PMC5910240

[88] Titze, B. , & Denk, W. (2013). Automated in‐chamber specimen coating for serial block‐face electron microscopy. Journal of Microscopy, 250(2), 101–10. 23451833 10.1111/jmi.12023

[89] Wanner, A. A. , Genoud, C. , Masudi, T. , Siksou, L. , & Friedrich, R. W. (2016). Dense EM‐based reconstruction of the interglomerular projectome in the zebrafish olfactory bulb. Nature Neuroscience, 19(6), 816–25. 27089019 10.1038/nn.4290

[90] Nguyen, H. B. , Thai, T. Q. , Saitoh, S. , Wu, B. , Saitoh, Y. , & Shimo, S. (2016). Conductive resins improve charging and resolution of acquired images in electron microscopic volume imaging. Scientific Reports, 6(1), 1–10. 27020327 10.1038/srep23721 PMC4810419

[91] Thai, T. Q. , Nguyen, H. B. , Saitoh, S. , Wu, B. , Saitoh, Y. , & Shimo, S. (2016). Rapid specimen preparation to improve the throughput of electron microscopic volume imaging for three‐dimensional analyses of subcellular ultrastructures with serial block‐face scanning electron microscopy. Medical Molecular Morphology, 49(3), 154–62. 26867664 10.1007/s00795-016-0134-7

[92] Heiligenstein, X. , De Beer, M. , Heiligenstein, J. , Eyraud, F. , Manet, L. , & Schmitt, F. (2021). HPM live μ for a full CLEM workflow. Methods in Cell Biology, 162, 115–49. 33707009 10.1016/bs.mcb.2020.10.022

[93] Guizar‐Sicairos, M. , Thurman, S. T. , & Fienup, J. R. (2008). Efficient subpixel image registration algorithms. Optics Letters, 33(2), 156–8. 18197224 10.1364/ol.33.000156

[94] Lowe, D. G. (1999). Object recognition from local scale‐invariant features. *Proceedings of the seventh IEEE International Conference on Computer Vision*, IEEE. vol. 2, p. 1150–7.

[95] Bay, H. , Ess, A. , Tuytelaars, T. , & Van Gool, L. (2008). Speeded‐up robust features (SURF). Computer Vision and Image Understanding, 110(3), 346–59.

[96] Fischler, M. A. , & Bolles, R. C. (1981). Random sample consensus: A paradigm for model fitting with applications to image analysis and automated cartography. Communications of the ACM, 24(6), 381–95.

[97] Anderson, J. R. , Jones, B. W. , Watt, C. B. , Shaw, M. V. , Yang, J.‐H. , & DeMill, D. (2011). Exploring the retinal connectome. Molecular Vision, 17, 355. 21311605 PMC3036568

[98] Thevenaz, P. , Ruttimann, U. E. , & Unser, M. (1998). A pyramid approach to subpixel registration based on intensity. IEEE Transactions on Image Processing, 7(1), 27–41. 10.1109/83.650848 18267377

[99] Khairy, K. , Denisov, G. , & Saalfeld, S. (2018) Joint deformable registration of large EM image volumes: A matrix solver approach. *ArXiv Preprint ArXiv:180410019*.

[100] Cardona, A. , Saalfeld, S. , Schindelin, J. , Arganda‐Carreras, I. , Preibisch, S. , & Longair, M. (2012). TrakEM2 software for neural circuit reconstruction. PLoS One, 7(6), e38011. 22723842 10.1371/journal.pone.0038011 PMC3378562

[101] Kremer, J. R. , Mastronarde, D. N. , & McIntosh, J. R. (1996). Computer visualization of three‐dimensional image data using IMOD. Journal of Structural Biology, 116(1), 71–6. 8742726 10.1006/jsbi.1996.0013

[102] Belevich, I. , Joensuu, M. , Kumar, D. , Vihinen, H. , & Jokitalo, E. (2016). Microscopy image browser: A platform for segmentation and analysis of multidimensional datasets. PLoS Biology, 14(1), e1002340. 26727152 10.1371/journal.pbio.1002340 PMC4699692

[103] Wetzel, A. W. , Bakal, J. , Dittrich, M. , Hildebrand, D. G. C. , Morgan, J. L. , & Lichtman, J. W. (2016). Registering large volume serial‐section electron microscopy image sets for neural circuit reconstruction using FFT signal whitening. 2016 IEEE Applied Imagery Pattern Recognition Workshop (AIPR), IEEE. p. 1–10.

[104] Saalfeld, S. , Cardona, A. , Hartenstein, V. , & Tomančák, P. (2009). CATMAID: Collaborative annotation toolkit for massive amounts of image data. Bioinformatics, 25(15), 1984–6. 19376822 10.1093/bioinformatics/btp266 PMC2712332

[105] Helmstaedter, M. , Briggman, K. L. , & Denk, W. (2011). High‐accuracy neurite reconstruction for high‐throughput neuroanatomy. Nature Neuroscience, 14(8), 1081–8. 21743472 10.1038/nn.2868

[106] Boergens, K. M. , Berning, M. , Bocklisch, T. , Bräunlein, D. , Drawitsch, F. , & Frohnhofen, J. (2017). webKnossos: Efficient online 3D data annotation for connectomics. Nature Methods, 14(7), 691–4. 28604722 10.1038/nmeth.4331

[107] Berger, D. R. , Seung, H. S. , & Lichtman, J. W. (2018). VAST (volume annotation and segmentation tool): Efficient manual and semi‐automatic labeling of large 3D image stacks. Frontiers in Neural Circuits, 12, 88. 30386216 10.3389/fncir.2018.00088 PMC6198149

[108] Kornfeld, J. , Svara, F. , & Wanner, A. A. (2020). Image processing for volume electron microscopy. In: Volume microscopy (p. 245–62). Springer.

[109] Jain, V. , Murray, J. F. , Roth, F. , Turaga, S. , Zhigulin, V. , & Briggman, K. L. (2007). Supervised learning of image restoration with convolutional networks. *2007 IEEE 11th International Conference on Computer Vision*, IEEE, p. 1–8.

[110] Arganda‐Carreras, I. , Turaga, S. C. , Berger, D. R. , Cireşan, D. , Giusti, A. , & Gambardella, L. M. (2015). Crowdsourcing the creation of image segmentation algorithms for connectomics. Frontiers in Neuroanatomy, 9, 142. 26594156 10.3389/fnana.2015.00142 PMC4633678

[111] Ronneberger, O. , Fischer, P. , & Brox, T. (2015). U‐net: Convolutional networks for biomedical image segmentation. *International Conference on Medical Image Computing and Computer‐Assisted Intervention*, Springer, p. 234–41.

[112] Çiçek, Ö. , Abdulkadir, A. , Lienkamp, S. S. , Brox, T. , & Ronneberger, O. (2016) 3D U‐Net: Learning dense volumetric segmentation from sparse annotation. *International Conference on Medical Image Computing and Computer‐Assisted Intervention*, Springer, p. 424–32.

[113] Quan, T. M. , Hildebrand, D. G. C. , & Jeong, W.‐K. (2016) Fusionnet: A deep fully residual convolutional neural network for image segmentation in connectomics. *ArXiv Preprint ArXiv:161205360*.

[114] Fakhry, A. , Zeng, T. , & Ji, S. (2016). Residual deconvolutional networks for brain electron microscopy image segmentation. IEEE Transactions on Medical Imaging, 36(2), 447–56. 28113967 10.1109/TMI.2016.2613019

[115] Zeng, T. , Wu, B. , & Ji, S. (2017). DeepEM3D: Approaching human‐level performance on 3D anisotropic EM image segmentation. Bioinformatics, 33(16), 2555–62. 28379412 10.1093/bioinformatics/btx188 PMC6248556

[116] Beier, T. , Pape, C. , Rahaman, N. , Prange, T. , Berg, S. , & Bock, D. D. (2017). Multicut brings automated neurite segmentation closer to human performance. Nature Methods, 14(2), 101–2. 28139671 10.1038/nmeth.4151

[117] Lee, K. , Zung, J. , Li, P. , Jain, V. , & Seung, H. S. (2017) Superhuman accuracy on the SNEMI3D connectomics challenge. *ArXiv Preprint ArXiv:170600120*.

[118] Funke, J. , Tschopp, F. , Grisaitis, W. , Sheridan, A. , Singh, C. , & Saalfeld, S. (2018). Large scale image segmentation with structured loss based deep learning for connectome reconstruction. IEEE Transactions on Pattern Analysis and Machine Intelligence, 41(7), 1669–80. 29993708 10.1109/TPAMI.2018.2835450

[119] Januszewski, M. , Maitin‐Shepard, J. , Li, P. , Kornfeld, J. , Denk, W. , & Jain, V. (2016) Flood‐filling networks. *ArXiv Preprint ArXiv:161100421*. 10.1038/s41592-018-0049-4 30013046

[120] Januszewski, M. , Kornfeld, J. , Li, P. H. , Pope, A. , Blakely, T. , & Lindsey, L. (2018). High‐precision automated reconstruction of neurons with flood‐filling networks. Nature Methods, 15(8), 605–10. 30013046 10.1038/s41592-018-0049-4

[121] Lee, K. , Turner, N. , Macrina, T. , Wu, J. , Lu, R. , & Seung, H. S. (2019). Convolutional nets for reconstructing neural circuits from brain images acquired by serial section electron microscopy. Current Opinion in Neurobiology, 55, 188–98. 31071619 10.1016/j.conb.2019.04.001 PMC6559369

[122] Kreshuk, A. , Straehle, C. N. , Sommer, C. , Koethe, U. , Cantoni, M. , & Knott, G. (2011). Automated detection and segmentation of synaptic contacts in nearly isotropic serial electron microscopy images. PLoS One, 6(10), e24899. 22031814 10.1371/journal.pone.0024899 PMC3198725

[123] Kreshuk, A. , Koethe, U. , Pax, E. , Bock, D. D. , & Hamprecht, F. A. (2014). Automated detection of synapses in serial section transmission electron microscopy image stacks. PLoS One, 9(2), e87351. 24516550 10.1371/journal.pone.0087351 PMC3916342

[124] Roncal, W. G. , Kaynig‐Fittkau, V. , Kasthuri, N. , Berger, D. , Vogelstein, J. T. , & Fernandez, L. R. (2014) Volumetric exploitation of synaptic information using context localization and evaluation. *ArXiv Preprint ArXiv:14033724*.

[125] Huang, G. B. , & Plaza, S. (2014) Identifying synapses using deep and wide multiscale recursive networks. *ArXiv Preprint ArXiv:14091789*.

[126] Dorkenwald, S. , Schubert, P. J. , Killinger, M. F. , Urban, G. , Mikula, S. , & Svara, F. (2017). Automated synaptic connectivity inference for volume electron microscopy. Nature Methods, 14(4), 435–42. 28250467 10.1038/nmeth.4206

[127] Santurkar, S. , Budden, D. , Matveev, A. , Berlin, H. , Saribekyan, H. , & Meirovitch, Y. (2017) Toward streaming synapse detection with compositional convnets. *ArXiv Preprint ArXiv:170207386*.

[128] Staffler, B. , Berning, M. , Boergens, K. M. , Gour, A. , der, S. P. v. , & Helmstaedter, M. (2017). SynEM, automated synapse detection for connectomics. Elife, 6, e26414. 28708060 10.7554/eLife.26414 PMC5658066

[129] Huang, G. B. , Scheffer, L. K. , & Plaza, S. M. (2018). Fully‐automatic synapse prediction and validation on a large data set. Frontiers in Neural Circuits, 12, 87. 30420797 10.3389/fncir.2018.00087 PMC6215860

[130] Heinrich, L. , Funke, J. , Pape, C. , Nunez‐Iglesias, J. , & Saalfeld, S. (2018) Synaptic cleft segmentation in non‐isotropic volume electron microscopy of the complete drosophila brain. *International Conference on Medical Image Computing and Computer‐Assisted Intervention*, Springer, p. 317–25.

[131] Buhmann, J. , Krause, R. , Lentini, R. C. , Eckstein, N. , Cook, M. , & Turaga, S. (2018) Synaptic partner prediction from point annotations in insect brains. *International Conference on Medical Image Computing and Computer‐Assisted Intervention*, Springer, p. 309–16.

[132] Buhmann, J. , Sheridan, A. , Malin‐Mayor, C. , Schlegel, P. , Gerhard, S. , & Kazimiers, T. (2021). Automatic detection of synaptic partners in a whole‐brain Drosophila electron microscopy data set. Nature Methods, 18(7), 771–774. 34168373 10.1038/s41592-021-01183-7 PMC7611460

[133] Spiers, H. , Songhurst, H. , Nightingale, L. , de, F. J. , Community, Z. V. , & Hutchings, R. (2021). Deep learning for automatic segmentation of the nuclear envelope in electron microscopy data, trained with volunteer segmentations. Traffic (Copenhagen, Denmark), 22(7), 240–253. 10.1111/tra.12789 33914396

[134] Briggman, K. L. , Helmstaedter, M. , & Denk, W. (2011). Wiring specificity in the direction‐selectivity circuit of the retina. Nature, 471(7337), 183–8. 21390125 10.1038/nature09818

[135] Kim, J. S. , Greene, M. J. , Zlateski, A. , Lee, K. , Richardson, M. , & Turaga, S. C. (2014). Space–time wiring specificity supports direction selectivity in the retina. Nature, 509(7500), 331–6. 24805243 10.1038/nature13240 PMC4074887

[136] Dorkenwald, S. , McKellar, C. E. , Macrina, T. , Kemnitz, N. , Lee, K. , Lu, R. , Wu, J. , Popovych, S. , Mitchell, E. , & Nehoran, B. (2021). Flywire: Online community for whole‐brain connectomics. Nature Methods, 19(1), 119–128. 10.1038/s41592-021-01330-0 34949809 PMC8903166

[137] Wei, D. , Lin, Z. , Franco‐Barranco, D. , Wendt, N. , Liu, X. , & Yin, W. (2020). Mitoem dataset: Large‐scale 3d mitochondria instance segmentation from EM images. *International Conference on Medical Image Computing and Computer‐Assisted Intervention*, Springer, p. 66–76. 10.1007/978-3-030-59722-1_7 PMC7713709 33283212

[138] Conrad, R. , & Narayan, K. (2021). CEM500K, a large‐scale heterogeneous unlabeled cellular electron microscopy image dataset for deep learning. Elife, 10, e65894. 33830015 10.7554/eLife.65894 PMC8032397

[139] Oztel, I. , Yolcu, G. , Ersoy, I. , White, T. , & Bunyak, F. (2017). Mitochondria segmentation in electron microscopy volumes using deep convolutional neural network. *2017 IEEE International Conference on Bioinformatics and Biomedicine (BIBM)*, IEEE, p. 1195–200.

[140] Mekuč, M. Ž. , Bohak, C. , Hudoklin, S. , Kim, B. H. , Kim, M. Y. , & Marolt, M. (2020). Automatic segmentation of mitochondria and endolysosomes in volumetric electron microscopy data. Computers in Biology and Medicine, 119, 103693. 32339123 10.1016/j.compbiomed.2020.103693

[141] Nightingale, L. , Folter, J. d. , Spiers, H. , Strange, A. , Collinson, L. M. , & Jones, M. L. (2021). Automatic instance segmentation of mitochondria in electron microscopy data. *BioRxiv*.

[142] Karabağ, C. , Jones, M. L. , Peddie, C. J. , Weston, A. E. , Collinson, L. M. , & Reyes‐Aldasoro, C. C. (2019). Segmentation and modelling of the nuclear envelope of hela cells imaged with serial block face scanning electron microscopy. Journal of Imaging, 5(9), 75. 34460669 10.3390/jimaging5090075 PMC8320948

[143] Karabağ, C. , Jones, M. L. , & Reyes‐Aldasoro, C. C. (2021). volumetric semantic instance segmentation of the plasma membrane of HeLa cells. Journal of Imaging, 7(6), 93. 10.3390/jimaging7060093 PMC8321355 39080881

[144] Zhang, X. , Peng, X. , Han, C. , Zhu, W. , Wei, L. , & Zhang, Y. (2019). A unified deep‐learning network to accurately segment insulin granules of different animal models imaged under different electron microscopy methodologies. Protein & Cell, 10(4), 306–11. 30306458 10.1007/s13238-018-0575-y PMC6418072

[145] Turaga, S. C. , Briggman, K. L. , Helmstaedter, M. , Denk, W. , & Seung, H. S. (2009) Maximin affinity learning of image segmentation. *ArXiv Preprint ArXiv:09115372*.

[146] Linsley, D. , Kim, J. , Berson, D. , & Serre, T. (2018) Robust neural circuit reconstruction from serial electron microscopy with convolutional recurrent networks. *ArXiv Preprint ArXiv:181111356*.

[147] Januszewski, M. , & Jain, V. (2019). Segmentation‐enhanced CycleGAN. bioRxiv, 48081.

[148] Roels, J. , Hennies, J. , Saeys, Y. , Philips, W. , & Kreshuk, A. (2019). Domain adaptive segmentation in volume electron microscopy imaging. *2019 IEEE 16th International Symposium on Biomedical Imaging (ISBI 2019)*, IEEE, p. 1519–22.

[149] Arganda‐Carreras, I. , Kaynig, V. , Rueden, C. , Eliceiri, K. W. , Schindelin, J. , & Cardona, A. (2017). Trainable Weka Segmentation: A machine learning tool for microscopy pixel classification. Bioinformatics, 33(15), 2424–6. 28369169 10.1093/bioinformatics/btx180

[150] Gómez‐de‐Mariscal, E. , García‐López‐de‐Haro, C. , Donati, L. , Unser, M. , Muñoz‐Barrutia, A. , & Sage, D. (2019). 7 DeepImageJ: A user‐friendly plugin to run deep learning models in ImageJ. Nature Methods, 18, 1192–1195. 10.1038/s41592-021-01262-9 34594030

[151] Berg, S. , Kutra, D. , Kroeger, T. , Straehle, C. N. , Kausler, B. X. , & Haubold, C. (2019). Ilastik: Interactive machine learning for (bio) image analysis. Nature Methods, 16, 1226–1232. 31570887 10.1038/s41592-019-0582-9

[152] Belevich, I. , & Jokitalo, E. (2021). DeepMIB: User‐friendly and open‐source software for training of deep learning network for biological image segmentation. PLoS Computational Biology, 17(3), e1008374. 33651804 10.1371/journal.pcbi.1008374 PMC7954287

[153] Urakubo, H. , Bullmann, T. , Kubota, Y. , Oba, S. , & Ishii, S. (2019). UNI‐EM: An environment for deep neural network‐based automated segmentation of neuronal electron microscopic images. Scientific Reports, 9(1), 1–9. 31857624 10.1038/s41598-019-55431-0 PMC6923391

[154] Haberl, M. G. , Churas, C. , Tindall, L. , Boassa, D. , Phan, S. , & Bushong, E. A. (2018). CDeep3M—Plug‐and‐Play cloud‐based deep learning for image segmentation. Nature Methods, 15(9), 677–80. 30171236 10.1038/s41592-018-0106-z PMC6548193

[155] von, C. L. , RF, L. , Jukkala, J. , Spahn, C. , Krentzel, D. , & Nehme, E. (2021). Democratising deep learning for microscopy with ZeroCostDL4Mic. Nature Communications, 12(1), 1–18. 10.1038/s41467-021-22518-0 PMC8050272 33859193

[156] Iudin, A. , Korir, P. K. , Salavert‐Torres, J. , Kleywegt, G. J. , & Patwardhan, A. (2016). EMPIAR: A public archive for raw electron microscopy image data. Nature Methods, 13(5), 387–8. 27067018 10.1038/nmeth.3806

[157] Williams, E. , Moore, J. , Li, S. W. , Rustici, G. , Tarkowska, A. , & Chessel, A. (2017). Image data resource: A bioimage data integration and publication platform. Nature Methods, 14(8), 775–81. 28775673 10.1038/nmeth.4326 PMC5536224

[158] Wilkinson, M. D. , Dumontier, M. , Aalbersberg, I. J. J. , Appleton, G. , Axton, M. , & Baak, A. (2016). The FAIR Guiding Principles for scientific data management and stewardship. Scientific Data, 3(1), 1–9. 10.1038/sdata.2016.18 PMC4792175 26978244

[159] Sarkans, U. , Chiu, W. , Collinson, L. , Darrow, M. C. , Ellenberg, J. , & Grunwald, D. (2021). REMBI: Recommended Metadata for Biological Images – Enabling reuse of microscopy data in biology. Nature Methods, 18(12), 1418–1422. 34021280 10.1038/s41592-021-01166-8 PMC8606015

[160] Moore, J. , Allan, C. , Besson, S. , Burel, J.‐M. , Diel, E. , & Gault, D. (2021) OME‐NGFF: Scalable format strategies for interoperable bioimaging data. *BioRxiv*. 10.1038/s41592-021-01326-w PMC8648559 34845388

[161] Zhao, T. , Olbris, D. J. , Yu, Y. , & Plaza, S. M. (2018). Neutu: Software for collaborative, large‐scale, segmentation‐based connectome reconstruction. Frontiers in Neural Circuits, 12, 101. 30483068 10.3389/fncir.2018.00101 PMC6243011

[162] Allan, C. , Burel, J.‐M. , Moore, J. , Blackburn, C. , Linkert, M. , & Loynton, S. (2012). OMERO: Flexible, model‐driven data management for experimental biology. Nature Methods, 9(3), 245–53. 22373911 10.1038/nmeth.1896 PMC3437820

[163] Linkert, M. , Rueden, C. T. , Allan, C. , Burel, J.‐M. , Moore, W. , & Patterson, A. (2010). Metadata matters: Access to image data in the real world. Journal of Cell Biology, 189(5), 777–82. 20513764 10.1083/jcb.201004104 PMC2878938

[164] Vogelstein, J. T. , Perlman, E. , Falk, B. , Baden, A. , Roncal, W. G. , & Chandrashekhar, V. (2018). A community‐developed open‐source computational ecosystem for big neuro data. Nature Methods, 15(11), 846–7. 30377345 10.1038/s41592-018-0181-1 PMC6481161

[165] Hider, R. , Kleissas, D. M. , Pryor, D. , Gion, T. , Rodriguez, L. , & Matelsky, J. (2019). The block object storage service (bossDB): A cloud‐native approach for petascale neuroscience discovery. *bioRxiv*. 10.1101/217745 PMC8885591 35242021

[166] Xu, C. S. , Pang, S. , Shtengel, G. , Müller, A. , Ritter, A. T. , & Hoffman, H. K. (2021). An open‐access volume electron microscopy atlas of whole cells and tissues. Nature, 599(7883), 147–151. 34616045 10.1038/s41586-021-03992-4 PMC9004664

[167] Vescovi, R. , Li, H. , Kinnison, J. , Keçeli, M. , Salim, M. , & Kasthuri, N. (2020) Toward an automated HPC pipeline for processing large scale electron microscopy data. *2020 IEEE/ACM 2nd Annual Workshop on Extreme‐scale Experiment‐in‐the‐Loop Computing (XLOOP)*, IEEE,p. 16–22.

[168] Katz, W. T. , & Plaza, S. M. (2019). DVID: Distributed versioned image‐oriented data service. Frontiers in Neural Circuits, 13, 5. 30804760 10.3389/fncir.2019.00005 PMC6371063

[169] Pietzsch, T. , Saalfeld, S. , Preibisch, S. , & Tomancak, P. (2015). BigDataViewer: Visualization and processing for large image data sets. Nature Methods, 12(6), 481–3. 26020499 10.1038/nmeth.3392

[170] Kume, S. (2021) Short review: Pathology of the image big data era using electron microscopy. *ArXiv Preprint ArXiv:211113627*.

[171] Cali, C. , Wawrzyniak, M. , Becker, C. , Maco, B. , Cantoni, M. , & Jorstad, A. (2018). The effects of aging on neuropil structure in mouse somatosensory cortex – A 3D electron microscopy analysis of layer 1. PLoS One, 13(7), e0198131. 29966021 10.1371/journal.pone.0198131 PMC6028106

[172] Gour, A. , Boergens, K. M. , Heike, N. , Hua, Y. , Laserstein, P. , & Song, K. (2021). Postnatal connectomic development of inhibition in mouse barrel cortex. Science, *371*(6528), https://www.science.org/doi/10.1126/science.abb4534 10.1126/science.abb4534 33273061

[173] Badea, A. , Ali‐Sharief, A. A. , & Johnson, G. A. (2007). Morphometric analysis of the C57BL/6J mouse brain. Neuroimage, 37(3), 683–93. 17627846 10.1016/j.neuroimage.2007.05.046 PMC2176152

[174] Zuidema, W. , & Kruit, P. (2020). Transmission imaging on a scintillator in a scanning electron microscope. Ultramicroscopy, 218, 113055. 32731131 10.1016/j.ultramic.2020.113055

[175] Titze, B. , & Genoud, C. (2016). Volume scanning electron microscopy for imaging biological ultrastructure. Biology of the Cell, 108(11), 307–23. 27432264 10.1111/boc.201600024

[176] Smith, D. , & Starborg, T. (2019). Serial block face scanning electron microscopy in cell biology: Applications and technology. Tissue and Cell, 57, 111–22. 30220487 10.1016/j.tice.2018.08.011

[177] White, J. G. , Southgate, E. , Thomson, J. N. , & Brenner, S. (1986). The structure of the nervous system of the nematode *Caenorhabditis elegans* . Philosophical Transactions of the Royal Society of London. Series B: Biological Sciences, 314(1165), 1–340. 22462104 10.1098/rstb.1986.0056

[178] Cook, S. J. , Jarrell, T. A. , Brittin, C. A. , Wang, Y. , Bloniarz, A. E. , & Yakovlev, M. A. (2019). Whole‐animal connectomes of both *Caenorhabditis elegans* sexes. Nature, 571(7763), 63–71. 31270481 10.1038/s41586-019-1352-7 PMC6889226

[179] Ryan, K. , Lu, Z. , & Meinertzhagen, I. A. (2016). The CNS connectome of a tadpole larva of *Ciona intestinalis* (L.) highlights sidedness in the brain of a chordate sibling. Elife, 5, e16962. 27921996 10.7554/eLife.16962 PMC5140270

[180] Ohyama, T. , Schneider‐Mizell, C. M. , Fetter, R. D. , Aleman, J. V. , Franconville, R. , & Rivera‐Alba, M. (2015). A multilevel multimodal circuit enhances action selection in Drosophila. Nature, 520(7549), 633–9. 25896325 10.1038/nature14297

[181] Takemura, S.‐Y. , Aso, Y. , Hige, T. , Wong, A. , Lu, Z. , & Xu, C. S. (2017). A connectome of a learning and memory center in the adult Drosophila brain. Elife, 6, e26975. 28718765 10.7554/eLife.26975 PMC5550281

[182] Vishwanathan, A. , Daie, K. , Ramirez, A. D. , Lichtman, J. W. , Aksay, E. R. F. , & Seung, H. S. (2017). Electron microscopic reconstruction of functionally identified cells in a neural integrator. Current Biology, 27(14), 2137–47. 28712570 10.1016/j.cub.2017.06.028 PMC5569574

[183] Guan, N. N. , Xu, L. , Zhang, T. , Huang, C.‐X. , Wang, Z. , & Dahlberg, E. (2021). A specialized spinal circuit for command amplification and directionality during escape behavior. Proceedings of the National Academy of Sciences, *118*(42). https://www.pnas.org/doi/10.1073/pnas.2106785118 10.1073/pnas.2106785118 PMC8545473 34663699

[184] Hua, Y. , Ding, X. , Wang, H. , Wang, F. , Lu, Y. , & Neef, J. (2021). Electron microscopic reconstruction of neural circuitry in the cochlea. Cell Reports, 34(1), 108551. 33406431 10.1016/j.celrep.2020.108551

[185] Tomassy, G. S. , Berger, D. R. , Chen, H. ‐H. , Kasthuri, N. , Hayworth, K. J. , & Vercelli, A. (2014). Distinct profiles of myelin distribution along single axons of pyramidal neurons in the neocortex. Science, 344(6181), 319–24. 24744380 10.1126/science.1249766 PMC4122120

[186] Meyer, C. , Mallouh, V. , Spehner, D. , Baudrier, E. , Schultz, P. , & Naegel, B. (2021) Automatic multi class organelle segmentation for cellular FIB‐SEM images. *2021 IEEE 18th International Symposium on Biomedical Imaging (ISBI)*, IEEE. pp. 668–72.

