# Supplementary material for: How innovations in methodology offer new prospects for volume electron microscopy
Source: J Microsc. 2022 Jul 27;287(3):114–37. doi: 10.1111/jmi.13134 (PMC9546337; doi:10.1111/jmi.13134)
Supplement: Supplementary file 1 — Supplementary Material [file JMI-287-114-s001.pdf]

SUPPLEMENTARY MATERIAL ACCOMPANYING THE MANUSCRIPT: "HOW INNOVATIONS IN METHODOLOGY OFFER NEW PROSPECTS FOR VOLUME ELECTRON MICROSCOPY"

Arent J. Kievits*, Ryan Lane, Elizabeth C. Carroll, Jacob P. Hoogenboom

Imaging Physics, Delft University of Technology, Delft, 2624CJ, The Netherlands

*Corresponding author: e-mail: A.J.Kievits@tudelft.nl

Table 1: List of volume EM studies and their biological application(s).

**Organism**	**Species / tissue type / cell line**	**Reference(s)**
Alga	*M. denticulata*	[1]
Arabidopsis (*A. thaliana*)	Root tips	[2]
	Roots	[3]
	Sieve element cells	[4]
Ascidian tadpole (*C. intestinalis*)	Larval CNS	[5]
Bacteria	*M. xanthus*	[6]
	B. subtilus	[7]
Nematode (*C. elegans*)	Larval hermaphrodite brain	[8]

	Adult hermaphrodite brain	[8]
	Adult male CNS	[9]
	Adult hermaphrodite CNS	[10]
Chick	Cornea	[11]
	Collagen fibrils	[12]
	Myocardium, endocardium, cardiac jelly	[13]
	Retina	[14]
Fruit fly (*D. melanogaster*)	Central brain	[15]
	Optic lobe	[16]
	Mushroom body	[17]
	Olfactory system	[18]
	Entire brain	[19]
	Visual system	[20]
	Optic Medulla	[21]
	Ventral nerve cord	[22]
	Trachael cells	[23]
	Ovarian follicular epithelium	[23]
	Larval sensory areas	[24]
	Larval brain neuropile, ventral nerve cord	[25]
	Larval CNS	[26]
G. lamblia trophozoite	Whole organism	[27]
Human	Cardiac Telocytes	[28]
	Lung epithelial cells	[2]
	Retinal pigment epithelium	[29]
	Immortalized Breast Cancer cells	[30,31]

	Immortalized T-Cell	[30,31]
	Macrophage	[30,31]
	Endothelial cells (HUVEC)	[32]
	Breast carcinoma	[33]
	Pancreatic adenocarcinoma	[33]
	Epidermal melanocytes	[34]
	Pancreatic carcinoid cells (BON)	[35]
	HeLa cells	[30,31]
	Hepatoma cells (Huh-7)	[36]
	Lung Alveolar Epithelium	[37]
	Connective tissue	[38]
	HIV-infected Primary CD4+ T cells	[39]
	HIV-infected macrophages	[40]
	HIV-infected dentritic cells and T cells	[41]
	Primary fetal astrocytes	[39]
	Jurkat CL.E6-1 cells	[39]
	Macrophages and *B. burgdorferi*	[42]
	Cerebral cortex	[43]
Barrelclover (*M. truncatula*)	Root tips	[2]
Monkey	COS-7 cell line	[44,31]
Mouse	Hippocampus	[45]
	Enteroendocrine cells	[46]
	Retinal starburst amacrine cells	[47,48]
	Primary somatosensory cortex layer 1	[49]
	Choroid Plexus	[50,31]

Lateral parietal association cortex	[51,52]
Retina inner plexiform layer	[53]
Cochlea (inner hair cells)	[54]
Posterior parietal cortex	[52]
Secondary visual cortex	[52]
Anterior cingulate cortex	[52]
Skin	[2]
Corpus callosum	[2]
Visual Thalamus	[55]
Primary beta cells	[56]
MD4 B cells	[57]
Pancreatic Islets	[58,59]
3T3 fibroblast cells	[60]
P. chabaudi infected erythrocytes	[61]
Liver	[62,63]
Optic nerve	[7]
Osteocyte lacuno-canalicular network	[64]
Urinary bladder	[65]
Visual cortex	[66,67,68]
Lung tissue	[2]
Cytotoxic T-Cell attacking cancer cell	[30]
Primary somatosensory cortex	[69]
Primary somatosensory cortex layer 4	[70,71]
Primary somatosensory cortex layer 2/3	[71]
Medial nucleus of the trapezoid body	[72]

	Neocortex	[73,74]
	Ventral Posteromedial Thalamic Nucleus	[75]
	Granule cells	[76]
	ventral tegmental area, Dopaminergic system	[77]
	Cerebellar cortex	[78]
Ringed worm (*P. dumerilii*)	Whole organism	[79]
Rabbit	Amacrine cell network	[80]
	Carotid artery elastin	[81]
	Retina	[82]
Rat	Astrocytes	[83,84]
	Cingulum	[85]
	Hepatocytes	[86]
	Corpus callosum	[85]
	Medial entorhinal cortex layer 2	[87]
	Perilesional cortex	[85]
	Podocytes	[88]
	Hippocampus	[89]
Sea urchin	Embryonic tissue	[90]
Sheep	Cardiomyocytes	[91]
Plankton	Thalassiosira pseudonana	[92]
Tobacco plant	Meiocytes	[93]
T. brucei	Full body	[94]
Yeast	Saccharomyces cerevisiae	[95]
Zebrafinch	High vocal center	[96]
Zebrafish (*D. rerio)*	Heart Junctional Region	[97]

Entire larval brain	[98]
Spinal segment	[99]
Larval spinal cord	[100]
Larval tail	[101]
Larval dorsal-lateral lanastomotic vessels	[102]
Larval intersegmental vessels	[102]
Larval Neuromasts	[103]
Larval olfactory bulb	[104,105]
Larval Hindbrain	[106]

References

1.Wanner G., Schäfer T., Lütz-Meindl U. 3-D analysis of dictyosomes and multivesicular bodies in the green alga Micrasterias denticulata by FIB/SEM tomography. *Journal of Structural Biology* 2013;**184**(2):203–11.

2.Kremer A., Lippens S., Bartunkova S., Asselbergh B., Blanpain C., Fendrych M., et al. Developing 3D SEM in a broad biological context. *Journal of Microscopy* 2015;**259**(2):80–96.

3.Scheuring D., Löfke C., Krüger F., Kittelmann M., Eisa A., Hughes L., et al. Actin-dependent vacuolar occupancy of the cell determines auxin-induced growth repression. *Proceedings of the National Academy of Sciences* 2016;**113**(2):452–7.

4.Furuta KM., Yadav SR., Lehesranta S., Belevich I., Miyashima S., Heo J-ok., et al. Arabidopsis NAC45/86 direct sieve element morphogenesis culminating in enucleation. *Science* 2014;**345**(6199):933–7.

5.Ryan K., Lu Z., Meinertzhagen IA. The CNS connectome of a tadpole larva of Ciona intestinalis

(L.) highlights sidedness in the brain of a chordate sibling. *Elife* 2016;**5**:e16962.

6.Remis JP., Wei D., Gorur A., Zemla M., Haraga J., Allen S., et al. Bacterial social networks: structure and composition of M yxococcus xanthus outer membrane vesicle chains. *Environmental Microbiology* 2014;**16**(2):598–610.

7.Schertel A., Snaidero N., Han H-M., Ruhwedel T., Laue M., Grabenbauer M., et al. Cryo FIB-SEM: volume imaging of cellular ultrastructure in native frozen specimens. *Journal of Structural Biology* 2013;**184**(2):355–60.

8.Witvliet D., Mulcahy B., Mitchell JK., Meirovitch Y., Berger DR., Wu Y., et al. Connectomes across development reveal principles of brain maturation. *Nature* 2021;**596**(7871):257–61.

9.Cook SJ., Jarrell TA., Brittin CA., Wang Y., Bloniarz AE., Yakovlev MA., et al. Whole-animal connectomes of both Caenorhabditis elegans sexes. *Nature* 2019;**571**(7763):63–71.

10.White JG., Southgate E., Thomson JN., Brenner S. The structure of the nervous system of the nematode Caenorhabditis elegans. *Philos Trans R Soc Lond B Biol Sci* 1986;**314**(1165):1–340.

11.Bushby AJ., P'ng KMY., Young RD., Pinali C., Knupp C., Quantock AJ. Imaging three-dimensional tissue architectures by focused ion beam scanning electron microscopy. *Nature Protocols* 2011;**6**(6):845–58.

12.Kalson NS., Starborg T., Lu Y., Mironov A., Humphries SM., Holmes DF., et al. Nonmuscle myosin II powered transport of newly formed collagen fibrils at the plasma membrane. *Proceedings of the National Academy of Sciences* 2013;**110**(49):E4743–E4752.

13.Rennie MY., Gahan CG., López CS., Thornburg KL., Rugonyi S. 3D imaging of the early embryonic chicken heart with focused ion beam scanning electron microscopy. *Microscopy and Microanalysis* 2014;**20**(4):1111–9.

14.Günther A., Dedek K., Haverkamp S., Irsen S., Briggman KL., Mouritsen H. Double cones

and the diverse connectivity of photoreceptors and bipolar cells in an avian retina. *Journal of Neuroscience* 2021;**41**(23):5015–28.

15.Scheffer LK., Xu CS., Januszewski M., Lu Z., Takemura S-ya., Hayworth KJ., et al. A Connectome and Analysis of the Adult Drosophila Central Brain. *BioRxiv* 2020.

16.Takemura S-ya., Xu CS., Lu Z., Rivlin PK., Parag T., Olbris DJ., et al. Synaptic circuits and their variations within different columns in the visual system of Drosophila. *Proceedings of the National Academy of Sciences* 2015;**112**(44):13711–6.

17.Takemura S-ya., Aso Y., Hige T., Wong A., Lu Z., Xu CS., et al. A connectome of a learning and memory center in the adult Drosophila brain. *Elife* 2017;**6**:e26975.

18.Tobin WF., Wilson RI., Lee W-CA. Wiring variations that enable and constrain neural computation in a sensory microcircuit. *Elife* 2017;**6**:e24838.

19.Zheng Z., Lauritzen JS., Perlman E., Robinson CG., Nichols M., Milkie D., et al. A complete electron microscopy volume of the brain of adult Drosophila melanogaster. *Cell* 2018;**174**(3):730–43.

20.Boergens KM., Kapfer C., Helmstaedter M., Denk W., Borst A. Full reconstruction of large lobula plate tangential cells in Drosophila from a 3D EM dataset. *Plos One* 2018;**13**(11):e0207828.

21.Takemura S-ya., Bharioke A., Lu Z., Nern A., Vitaladevuni S., Rivlin PK., et al. A visual motion detection circuit suggested by Drosophila connectomics. *Nature* 2013;**500**(7461):175–81.

22.Phelps JS., Hildebrand DGC., Graham BJ., Kuan AT., Thomas LA., Nguyen TM., et al. Reconstruction of motor control circuits in adult Drosophila using automated transmission electron microscopy. *Cell* 2021;**184**(3):759–74.

23.Ronchi P., Mizzon G., Machado P., D'Imprima E., Best BT., Cassella L., et al. High-precision targeting workflow for volume electron microscopy. *Journal of Cell Biology*

2021;**220**(9):e202104069.

24.Gerhard S., Andrade I., Fetter RD., Cardona A., Schneider-Mizell CM. Conserved neural circuit structure across Drosophila larval development revealed by comparative connectomics. *Elife* 2017;**6**:e29089.

25.Cardona A., Saalfeld S., Preibisch S., Schmid B., Cheng A., Pulokas J., et al. An integrated micro-and macroarchitectural analysis of the Drosophila brain by computer-assisted serial section electron microscopy. *PLoS Biology* 2010;**8**(10):e1000502.

26.Ohyama T., Schneider-Mizell CM., Fetter RD., Aleman JV., Franconville R., Rivera-Alba M., et al. A multilevel multimodal circuit enhances action selection in Drosophila. *Nature* 2015;**520**(7549):633–9.

27.Schwartz CL., Heumann JM., Dawson SC., Hoenger A. A detailed, hierarchical study of Giardia lamblia's ventral disc reveals novel microtubule-associated protein complexes 2012.

28.Cretoiu D., Hummel E., Zimmermann H., Gherghiceanu M., Popescu LM. Human cardiac telocytes: 3D imaging by FIB-SEM tomography. *Journal of Cellular and Molecular Medicine* 2014;**18**(11):2157–64.

29.Pollreisz A., Messinger JD., Sloan KR., Mittermueller TJ., Weinhandl AS., Benson EK., et al. Visualizing melanosomes, lipofuscin, and melanolipofuscin in human retinal pigment epithelium using serial block face scanning electron microscopy. *Experimental Eye Research* 2018;**166**:131–9.

30.Xu CS., Pang S., Hayworth KJ., Hess HF. Transforming fib-sem systems for large-volume connectomics and cell biology. *Volume Microscopy*, Springer; 2020, p. 221–43.

31.Heinrich L., Bennett D., Ackerman D., Park W., Bogovic J., Eckstein N., et al. Whole-cell organelle segmentation in volume electron microscopy. *Nature* 2021:1–6.

32.Arévalo MT., Simpson-Haidaris PJ., Kou Z., Schlesinger JJ., Jin X. Primary human endothelial cells support direct but not antibody-dependent enhancement of dengue viral infection. *Journal of Medical Virology* 2009;**81**(3):519–28.

33.Machireddy A., Thibault G., Loftis KG., Stoltz K., Bueno CE., Smith HR., et al. Robust Segmentation of Cellular Ultrastructure on Sparsely Labeled 3D Electron Microscopy Images using Deep Learning. *Available at SSRN 3830021* 2021.

34.Mun JY., Jeong SY., Kim JH., Han SS., Kim I-H. A low fluence Q-switched Nd: YAG laser modifies the 3D structure of melanocyte and ultrastructure of melanosome by subcellular-selective photothermolysis. *Journal of Electron Microscopy* 2010;**60**(1):11–8.

35.Villinger C., Gregorius H., Kranz C., Höhn K., Münzberg C., Wichert G von., et al. FIB/SEM tomography with TEM-like resolution for 3D imaging of high-pressure frozen cells. *Histochemistry and Cell Biology* 2012;**138**(4):549–56.

36.Vihinen H., Belevich I., Jokitalo E. Three dimensional electron microscopy of cellular organelles by serial block face SEM and ET. *Microsc Anal* 2013;**27**:7–10.

37.Schneider JP., Wrede C., Mühlfeld C. The three-dimensional ultrastructure of the human alveolar epithelium revealed by focused ion beam electron microscopy. *International Journal of Molecular Sciences* 2020;**21**(3):1089.

38.Svensson RB., Herchenhan A., Starborg T., Larsen M., Kadler KE., Qvortrup K., et al. Evidence of structurally continuous collagen fibrils in tendons. *Acta Biomaterialia* 2017;**50**:293–301.

39.Do T., Murphy G., Earl LA., Del Prete GQ., Grandinetti G., Li G-H., et al. Three-dimensional imaging of HIV-1 virological synapses reveals membrane architectures involved in virus transmission. *Journal of Virology* 2014;**88**(18):10327–39.

40.Bennett AE., Narayan K., Shi D., Hartnell LM., Gousset K., He H., et al. Ion-abrasion scanning

electron microscopy reveals surface-connected tubular conduits in HIV-infected macrophages. *PLoS Pathogens* 2009;**5**(9):e1000591.

41.Felts RL., Narayan K., Estes JD., Shi D., Trubey CM., Fu J., et al. 3D visualization of HIV transfer at the virological synapse between dendritic cells and T cells. *Proceedings of the National Academy of Sciences* 2010;**107**(30):13336–41.

42.Klose M., Scheungrab M., Luckner M., Wanner G., Linder S. FIB-SEM-based analysis of Borrelia intracellular processing by human macrophages. *Journal of Cell Science* 2021;**134**(5):jcs252320.

43.Shapson-Coe A., Januszewski M., Berger DR., Pope A., Wu Y., Blakely T., et al. A connectomic study of a petascale fragment of human cerebral cortex. *BioRxiv* 2021.

44.Hoffman DP., Shtengel G., Xu CS., Campbell KR., Freeman M., Wang L., et al. Correlative three-dimensional super-resolution and block-face electron microscopy of whole vitreously frozen cells. *Science* 2020;**367**(6475).

45.Bloss EB., Cembrowski MS., Karsh B., Colonell J., Fetter RD., Spruston N. Single excitatory axons form clustered synapses onto CA1 pyramidal cell dendrites. *Nature Neuroscience* 2018;**21**(3):353–63.

46.Bohórquez DV., Samsa LA., Roholt A., Medicetty S., Chandra R., Liddle RA. An enteroendocrine cell–enteric glia connection revealed by 3D electron microscopy. *PloS One* 2014;**9**(2):e89881.

47.Briggman KL., Helmstaedter M., Denk W. Wiring specificity in the direction-selectivity circuit of the retina. *Nature* 2011;**471**(7337):183–8.

48.Ding H., Smith RG., Poleg-Polsky A., Diamond JS., Briggman KL. Species-specific wiring for direction selectivity in the mammalian retina. *Nature* 2016;**535**(7610):105–10.

49.Cali C., Wawrzyniak M., Becker C., Maco B., Cantoni M., Jorstad A., et al. The effects of aging on neuropil structure in mouse somatosensory cortex—A 3D electron microscopy analysis of layer 1. *PLoS One* 2018;**13**(7):e0198131.

50.Coulter ME., Dorobantu CM., Lodewijk GA., Delalande F., Cianférani S., Ganesh VS., et al. The ESCRT-III protein CHMP1A mediates secretion of sonic hedgehog on a distinctive subtype of extracellular vesicles. *Cell Reports* 2018;**24**(4):973–86.

51.Drawitsch F., Karimi A., Boergens KM., Helmstaedter M. FluoEM, virtual labeling of axons in three-dimensional electron microscopy data for long-range connectomics. *Elife* 2018;**7**:e38976.

52.Karimi A., Odenthal J., Drawitsch F., Boergens KM., Helmstaedter M. Cell-type specific innervation of cortical pyramidal cells at their apical dendrites. *Elife* 2020;**9**:e46876.

53.Helmstaedter M., Briggman KL., Turaga SC., Jain V., Seung HS., Denk W. Connectomic reconstruction of the inner plexiform layer in the mouse retina. *Nature* 2013;**500**(7461):168–74.

54.Hua Y., Ding X., Wang H., Wang F., Lu Y., Neef J., et al. Electron Microscopic Reconstruction of Neural Circuitry in the Cochlea. *Cell Reports* 2021;**34**(1):108551.

55.Morgan JL., Berger DR., Wetzel AW., Lichtman JW. The fuzzy logic of network connectivity in mouse visual thalamus. *Cell* 2016;**165**(1):192–206.

56.Müller A., Schmidt D., Xu CS., Pang S., D'Costa JV., Kretschmar S., et al. 3D FIB-SEM reconstruction of microtubule–organelle interaction in whole primary mouse β cells. *Journal of Cell Biology* 2020;**220**(2).

57.Thaunat O., Granja AG., Barral P., Filby A., Montaner B., Collinson L., et al. Asymmetric segregation of polarized antigen on B cell division shapes presentation capacity. *Science* 2012;**335**(6067):475–9.

58.Pfeifer CR., Shomorony A., Aronova MA., Zhang G., Cai T., Xu H., et al. Quantitative analysis

of mouse pancreatic islet architecture by serial block-face SEM. *Journal of Structural Biology* 2015;**189**(1):44–52.

59.Rao A., McBride EL., Zhang G., Xu H., Cai T., Notkins AL., et al. Determination of secretory granule maturation times in pancreatic islet β-cells by serial block-face electron microscopy. *Journal of Structural Biology* 2020;**212**(1):107584.

60.Kopek BG., Shtengel G., Xu CS., Clayton DA., Hess HF. Correlative 3D superresolution fluorescence and electron microscopy reveal the relationship of mitochondrial nucleoids to membranes. *Proceedings of the National Academy of Sciences* 2012;**109**(16):6136–41.

61.Soares Medeiros LC., De Souza W., Jiao C., Barrabin H., Miranda K. Visualizing the 3D architecture of multiple erythrocytes infected with Plasmodium at nanoscale by focused ion beam-scanning electron microscopy. *PLoS One* 2012;**7**(3):e33445.

62.Murphy GE., Lowekamp BC., Zerfas PM., Chandler RJ., Narasimha R., Venditti CP., et al. Ion-abrasion scanning electron microscopy reveals distorted liver mitochondrial morphology in murine methylmalonic acidemia. *Journal of Structural Biology* 2010;**171**(2):125–32.

63.Parlakgul G., Arruda AP., Cagampan E., Pang S., Guney E., Lee Y., et al. High resolution 3D imaging of liver reveals a central role for subcellular architectural organization in metabolism. *BioRxiv* 2020.

64.Schneider P., Meier M., Wepf R., Müller R. Serial FIB/SEM imaging for quantitative 3D assessment of the osteocyte lacuno-canalicular network. *Bone* 2011;**49**(2):304–11.

65.Mekuč MŽ., Bohak C., Hudoklin S., Kim BH., Kim MY., Marolt M., et al. Automatic segmentation of mitochondria and endolysosomes in volumetric electron microscopy data. *Computers in Biology and Medicine* 2020;**119**:103693.

66.Bock DD., Lee W-CA., Kerlin AM., Andermann ML., Hood G., Wetzel AW., et al. Network

anatomy and in vivo physiology of visual cortical neurons. *Nature* 2011;**471**(7337):177–82.

67.Lee W-CA., Bonin V., Reed M., Graham BJ., Hood G., Glattfelder K., et al. Anatomy and function of an excitatory network in the visual cortex. *Nature* 2016;**532**(7599):370–4.

68.Bae JA., Baptiste M., Bodor AL., Brittain D., Buchanan JA., Bumbarger DJ., et al. Functional connectomics spanning multiple areas of mouse visual cortex. *BioRxiv* 2021.

69.Tomassy GS., Berger DR., Chen H-H., Kasthuri N., Hayworth KJ., Vercelli A., et al. Distinct profiles of myelin distribution along single axons of pyramidal neurons in the neocortex. *Science* 2014;**344**(6181):319–24.

70.Motta A., Berning M., Boergens KM., Staffler B., Beining M., Loomba S., et al. Dense connectomic reconstruction in layer 4 of the somatosensory cortex. *Science* 2019;**366**(6469).

71.Gour A., Boergens KM., Heike N., Hua Y., Laserstein P., Song K., et al. Postnatal connectomic development of inhibition in mouse barrel cortex. *Science* 2021;**371**(6528).

72.Holcomb PS., Hoffpauir BK., Hoyson MC., Jackson DR., Deerinck TJ., Marrs GS., et al. Synaptic inputs compete during rapid formation of the calyx of Held: a new model system for neural development. *Journal of Neuroscience* 2013;**33**(32):12954–69.

73.Kasthuri N., Hayworth KJ., Berger DR., Schalek RL., Conchello JA., Knowles-Barley S., et al. Saturated reconstruction of a volume of neocortex. *Cell* 2015;**162**(3):648–61.

74.Yin W., Brittain D., Borseth J., Scott ME., Williams D., Perkins J., et al. A petascale automated imaging pipeline for mapping neuronal circuits with high-throughput transmission electron microscopy. *Nature Communications* 2020;**11**(1):1–12.

75.Rodriguez-Moreno J., Rollenhagen A., Arlandis J., Santuy A., Merchan-Pérez A., DeFelipe J., et al. Quantitative 3D ultrastructure of thalamocortical synapses from the “lemniscal” ventral posteromedial nucleus in mouse barrel cortex. *Cerebral Cortex* 2018;**28**(9):3159–75.

76.Bosch C., Martínez A., Masachs N., Teixeira CM., Fernaud I., Ulloa F., et al. FIB/SEM technology and high-throughput 3D reconstruction of dendritic spines and synapses in GFP-labeled adult-generated neurons. *Frontiers in Neuroanatomy* 2015;**9**:60.

77.Wildenberg G., Sorokina A., Koranda J., Monical A., Heer C., Sheffield M., et al. Partial connectomes of labeled dopaminergic circuits reveal non-synaptic communication and axonal remodeling after exposure to cocaine. *Elife* 2021;**10**:e71981.

78.Wilson AM., Schalek R., Suissa-Peleg A., Jones TR., Knowles-Barley S., Pfister H., et al. Developmental rewiring between cerebellar climbing fibers and Purkinje cells begins with positive feedback synapse addition. *Cell Reports* 2019;**29**(9):2849–61.

79.Vergara HM., Pape C., Meechan KI., Zinchenko V., Genoud C., Wanner AA., et al. Whole-body integration of gene expression and single-cell morphology. *Cell* 2021;**184**(18):4819–37.

80.Marc RE., Anderson JR., Jones BW., Sigulinsky CL., Lauritzen JS. The AII amacrine cell connectome: a dense network hub. *Frontiers in Neural Circuits* 2014;**8**:104.

81.Rezakhaniha R., Fonck E., Genoud C., Stergiopulos N. Role of elastin anisotropy in structural strain energy functions of arterial tissue. *Biomechanics and Modeling in Mechanobiology* 2011;**10**(4):599–611.

82.Anderson JR., Jones BW., Watt CB., Shaw MV., Yang J-H., DeMill D., et al. Exploring the retinal connectome. *Molecular Vision* 2011;**17**:355.

83.Calì C., Agus M., Kare K., Boges DJ., Lehväslaiho H., Hadwiger M., et al. 3D cellular reconstruction of cortical glia and parenchymal morphometric analysis from Serial Block-Face Electron Microscopy of juvenile rat. *Progress in Neurobiology* 2019;**183**:101696.

84.Kikuchi T., Gonzalez-Soriano J., Kastanauskaite A., Benavides-Piccione R., Merchan-Perez A., DeFelipe J., et al. Volume electron microscopy study of the relationship between synapses and

astrocytes in the developing rat somatosensory cortex. *Cerebral Cortex* 2020;**30**(6):3800–19.

85.Salo RA., Belevich I., Manninen E., Jokitalo E., Gröhn O., Sierra A. Quantification of anisotropy and orientation in 3D electron microscopy and diffusion tensor imaging in injured rat brain. *Neuroimage* 2018;**172**:404–14.

86.Rouquette J., Genoud C., Vazquez-Nin GH., Kraus B., Cremer T., Fakan S. Revealing the high-resolution three-dimensional network of chromatin and interchromatin space: a novel electron-microscopic approach to reconstructing nuclear architecture. *Chromosome Research* 2009;**17**(6):801–10.

87.Schmidt H., Gour A., Straehle J., Boergens KM., Brecht M., Helmstaedter M. Axonal synapse sorting in medial entorhinal cortex. *Nature* 2017;**549**(7673):469–75.

88.Ichimura K., Miyazaki N., Sadayama S., Murata K., Koike M., Nakamura K-ichiro., et al. Three-dimensional architecture of podocytes revealed by block-face scanning electron microscopy. *Scientific Reports* 2015;**5**(1):1–7.

89.Mishchenko Y., Hu T., Spacek J., Mendenhall J., Harris KM., Chklovskii DB. Ultrastructural analysis of hippocampal neuropil from the connectomics perspective. *Neuron* 2010;**67**(6):1009–20.

90.Vidavsky N., Addadi S., Schertel A., Ben-Ezra D., Shpigel M., Addadi L., et al. Calcium transport into the cells of the sea urchin larva in relation to spicule formation. *Proceedings of the National Academy of Sciences* 2016;**113**(45):12637–42.

91.Pinali C., Bennett H., Davenport JB., Trafford AW., Kitmitto A. Three-dimensional reconstruction of cardiac sarcoplasmic reticulum reveals a continuous network linking transverse-tubules: this organization is perturbed in heart failure. *Circulation Research* 2013;**113**(11):1219–30.

92.Scott K. 3D elemental and structural analysis of biological specimens using electrons and ions.

Journal of Microscopy 2011;**242**(1):86–93.

93.Mursalimov S., Ohno N., Matsumoto M., Bayborodin S., Deineko E. Serial Block-Face Scanning Electron Microscopy Reveals That Intercellular Nuclear Migration Occurs in Most Normal Tobacco Male Meiocytes. *Frontiers in Plant Science* 2021;**12**:775.

94.Hughes L., Borrett S., Towers K., Starborg T., Vaughan S. Patterns of organelle ontogeny through a cell cycle revealed by whole-cell reconstructions using 3D electron microscopy. *Journal of Cell Science* 2017;**130**(3):637–47.

95.Wei D., Jacobs S., Modla S., Zhang S., Young CL., Cirino R., et al. High-resolution three-dimensional reconstruction of a whole yeast cell using focused-ion beam scanning electron microscopy. *Biotechniques* 2012;**53**(1):41–8.

96.Kornfeld J., Benezra SE., Narayanan RT., Svara F., Egger R., Oberlaender M., et al. EM connectomics reveals axonal target variation in a sequence-generating network. *Elife* 2017;**6**:e24364.

97.Lafontant PJ., Behzad AR., Brown E., Landry P., Hu N., Burns AR. Cardiac myocyte diversity and a fibroblast network in the junctional region of the zebrafish heart revealed by transmission and serial block-face scanning electron microscopy. *PloS One* 2013;**8**(8):e72388.

98.Hildebrand DGC., Cicconet M., Torres RM., Choi W., Quan TM., Moon J., et al. Whole-brain serial-section electron microscopy in larval zebrafish. *Nature* 2017;**545**(7654):345–9.

99.Guan NN., Xu L., Zhang T., Huang C-X., Wang Z., Dahlberg E., et al. A specialized spinal circuit for command amplification and directionality during escape behavior. *Proceedings of the National Academy of Sciences* 2021;**118**(42).

100.Svara FN., Kornfeld J., Denk W., Bollmann JH. Volume EM reconstruction of spinal cord reveals wiring specificity in speed-related motor circuits. *Cell Reports* 2018;**23**(10):2942–54.

101.Vidavsky N., Akiva A., Kaplan-Ashiri I., Rechav K., Addadi L., Weiner S., et al. Cryo-FIB-

SEM serial milling and block face imaging: Large volume structural analysis of biological tissues preserved close to their native state. *Journal of Structural Biology* 2016;**196**(3):487–95.

102.Armer HEJ., Mariggi G., Png KMY., Genoud C., Monteith AG., Bushby AJ., et al. Imaging transient blood vessel fusion events in zebrafish by correlative volume electron microscopy. *PLoS One* 2009;**4**(11):e7716.

103.Dow E., Jacobo A., Hossain S., Siletti K., Hudspeth AJ. Connectomics of the zebrafish's lateral-line neuromast reveals wiring and miswiring in a simple microcircuit. *Elife* 2018;**7**:e33988.

104.Friedrich R., Genoud C., Wanner AA. Analyzing the structure and function of neuronal circuits in zebrafish. *Frontiers in Neural Circuits* 2013;**7**:71.

105.Wanner AA., Genoud C., Masudi T., Siksou L., Friedrich RW. Dense EM-based reconstruction of the interglomerular projectome in the zebrafish olfactory bulb. *Nature Neuroscience* 2016;**19**(6):816–25.

106.Vishwanathan A., Daie K., Ramirez AD., Lichtman JW., Aksay ERF., Seung HS. Electron microscopic reconstruction of functionally identified cells in a neural integrator. *Current Biology* 2017;**27**(14):2137–47.
